# *Cissus verticillata* Leaf Extract Decreases the Production of AGEs and ROS In Vitro

**DOI:** 10.3390/molecules31040697

**Published:** 2026-02-17

**Authors:** Felipe Nunes Cardoso, Emanuel Victor dos Santos Nunes, Ingrid Delbone Figueiredo, Winner Duque Rodrigues, Renata Pires Assis, André Gonzaga dos Santos, Luis Vitor Silva do Sacramento, Iguatemy Lourenço Brunetti, Alan Cesar Pilon, Amanda Martins Baviera

**Affiliations:** 1 Department of Clinical Analysis, School of Pharmaceutical Sciences, São Paulo State University (UNESP), Araraquara 14800-903, SP, Brazil; felipe.n.cardoso@unesp.br (F.N.C.); ingrid.delbone@unesp.br (I.D.F.); renatapires_17@hotmail.com (R.P.A.); iguatemy.brunetti@unesp.br (I.L.B.); 2 Nucleous of Bioassay, Biosynthesis and Ecophysiology of Natural Products, Institute of Chemistry, São Paulo State University (UNESP), Araraquara 14800-060, SP, Brazil; ev.nunes@unesp.br (E.V.d.S.N.); alan.pilon@unesp.br (A.C.P.); 3 Department of Drugs and Medicines, School of Pharmaceutical Sciences, São Paulo State University (UNESP), Araraquara 14800-903, SP, Brazil; winner.rodrigues@unesp.br (W.D.R.); andre.gonzaga@unesp.br (A.G.d.S.); luis.vitor@unesp.br (L.V.S.d.S.)

**Keywords:** medicinal plants, polyphenols, diabetes mellitus, in vitro model systems, protein glycation, glycoxidative stress

## Abstract

*Cissus verticillata* (plant-insulin) is used in the Brazilian popular medicine to treat symptoms of diabetes. Studies about its ability to contrast glycoxidative stress is lacking, which may add mechanistic information about its effects on treat diabetic complications. This study investigated the ability of *Cissus verticillata* leaf hydroethanolic extract (CvExt) to scavenge reactive oxygen species (ROS) and to inhibit the formation of advanced glycation end products (AGEs). ROS scavenging assays were used to test CvExt antioxidant activity. Incubations of bovine serum albumin with glucose (0.5 M) or methylglyoxal (2 mM) and CvExt (250, 125, and 62.5 μg/mL) were used to test the antiglycation activity, by monitoring fluorescent AGEs, markers of amino acid oxidation, and protein carbonyl groups (PCO). The plant extract was submitted to liquid−liquid extractions, fractions were analyzed by liquid chromatography with tandem mass spectrometry, and the data obtained were subjected to partial least-squares discriminant analysis. CvExt scavenged ROS inhibited the formation of AGEs and amino acid oxidation products, and decreased PCO levels. The main metabolites found in CvExt were flavonoids, cinnamic acid derivatives, coumarins, free amino acids, and some lipophilic compounds. CvExt inhibited glycoxidative stress in vitro, which can be associated with its complex chemical composition.

## 1. Introduction

Chronic hyperglycemia, a main feature of diabetes mellitus (DM), participates in the onset of various microvascular and macrovascular complications that are related to the increased rates of morbidity and mortality of this disease [1]. The pathogenesis of the diabetic complications is associated with the glycoxidation process, characterized by the exacerbated formation of advanced glycation end products (AGEs) and reactive oxygen species (ROS) beyond the capacities of the endogenous detoxification components [2,3].

During the glycoxidation process, highly reactive metabolites are formed such as the dicarbonyl compounds (α-oxoaldehydes) including glyoxal, methylglyoxal (MG), and 3-deoxyglucosone, which are mostly derived from the degradation of intermediates of the glycolytic pathway and lipid peroxidation. These dicarbonyl compounds act as potent glycating agents of biomolecules and generate a great variety of AGEs [4,5]. In diabetes, AGEs and ROS lead to extensive post-translational modifications and loss of function of proteins (including crosslink formation), lipid peroxidation, DNA strand breaks, damage of cellular machinery, and increased insulin resistance. In addition, by activating the RAGE receptor, AGEs also exacerbate tissue injuries by causing oxidative stress and pro-inflammatory effects [6,7].

Despite the vast range of currently available glucose-lowering drugs for DM management [8,9], there is still great interest from patients in the use of preparations or bioactive compounds of natural origin. In this context, there are growing efforts from the scientific community in the search of the antidiabetic activity of natural products. Natural bioactives have the ability to inhibit oxidative stress, protein glycation, and the formation of AGEs, and they can activate endogenous defense systems that detoxify AGEs and ROS. Therefore, they become promising candidates for complementary therapies targeting diabetic complications resulting from glycoxidative stress [10,11].

*Cissus verticillata* (L.) Nicolson & C.E. Jarvis is a plant species belonging to the Vitaceae family. It has a wide neotropical distribution, being found in Brazilian phytogeographic domains of the Amazon, Caatinga, Cerrado, Atlantic Forest, and Pantanal. In Brazil, it is popularly known as “anil-trepador”, “cipó-pucá”, and “uva-brava” [12]. *C. verticillata* preparations (mainly tea and decoction of leaves) have been used in the popular medicine to treat several illnesses, including respiratory diseases, hepatic and renal disorders, hypertension, and strokes [13,14]. In addition, in Brazilian popular medicine, the main use of preparations from *C. verticillata* leaves is for the treatment of DM, explaining the reason why this plant species is also known as vegetable-insulin (plant-insulin) [15].

Previous studies by our group have demonstrated the antidiabetic activity of *C. verticillata* in an in vivo model system of DM. For 30 days, streptozotocin-induced diabetic rats received a decoction of *C. verticillata* leaves (150 g of leaves/liter of water) daily in place of the drinking water. The treatment with *C. verticillata* promoted a significant decrease in the glycemia and glycosuria, as well as alleviated diabetic symptoms, including polyuria, polydipsia, and polyphagia [16]. However, as far as we know, there is still no evidence about the potential of *C. verticillata* to mitigate glycoxidative stress, which may add mechanistic information about the pharmacological activity of this species in an attempt to reinforce its beneficial effects on the treatment of DM and its complications.

By using in vitro model systems of protein glycation and ROS generation, the present study investigated the ability of *C. verticillata* leaf hydroethanolic extract (CvExt) to scavenge ROS and to inhibit AGE formation, establishing a correlation with the metabolites detected by liquid chromatography−tandem mass spectrometry (LC-MS/MS) and subsequent application of molecular networking techniques, associated with partial least-squares discriminant analysis (PLS-DA).

## 2. Results

### 2.1. In Vitro ROO^•^ and O_2_^•−^ Scavenging Assays

By using in vitro model systems of ROS generation, it was possible to investigate the antioxidant activity of *C. verticillata* leaf extract based on its ability to scavenge both superoxide anion radical (O_2_^•−^) and peroxyl radical (ROO^•^). The antioxidant capacity of CvExt and standards antioxidants (quercetin and Trolox) was performed by the analysis of the rate of the crocin bleaching inhibition (ROO^•^ scavenging assay), comparing the slope values for CvExt, quercetin, and Trolox. The order of decreasing antioxidant capacity was the following: quercetin ≥ Trolox > CvExt (Figure S1). In addition, the antioxidant capacity was also evaluated by determining the percentage of inhibition of the crocin bleaching and thus the EC_50_, which allows obtaining the effective concentration that inhibited 50% of the crocin bleaching (Table 1). The EC_50_ values for quercetin, Trolox, and CvExt follow the same order of efficiency as the slope values, where quercetin was slightly better than Trolox that was better than CvExt (Table 1).

The NBT reduction (O_2_^•−^ scavenging assay) was also evaluated by the percentage of inhibition by CvExt and the standards antioxidants, thus expressing the antioxidant capacity against O_2_^•−^. CvExt showed an in vitro antioxidant activity lower than that observed for quercetin; however, CvExt had capacity to capture O_2_^•−^ comparable to Trolox (Figure S2, Table 1).

### 2.2. In Vitro Assays for the Antiglycation Activity

**Figure 1:**
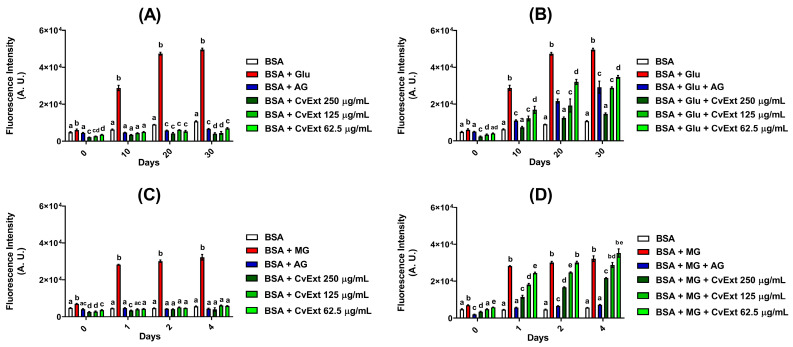
Effects of CvExt on the formation of fluorescent AGEs using in vitro model systems of protein glycation based on BSA + glucose (**A**,**B**) or BSA + MG (**C**,**D**). (**A**) Incubations with BSA for 30 days; (**B**) incubations with BSA + glucose for 30 days; (**C**) incubations with BSA for 4 days; (**D**) incubations with BSA + MG for 4 days. CvExt—*Cissus verticillata* leaf hydroethanolic extract; BSA—bovine serum albumin; Glu—glucose; MG—methylglyoxal; AG—aminoguanidine. Values are expressed as the mean ± SEM. Differences between groups were considered significant if *p* < 0.05 and were analyzed using one-way ANOVA followed by the Student−Newman−Keuls test. Means not sharing a common letter indicate significant differences.

By incubating bovine serum albumin (BSA) with glucose during 10, 20, and 30 days, the levels of fluorescent AGEs were increased by 4.8-fold, 7.9-fold, and 8.2-fold, respectively, when compared with the corresponding values of BSA alone (Figure 1A,B). For incubations of BSA + MG, the increases in the levels of fluorescent AGEs were 4-fold (1 day), 4.3-fold (2 days), and 4.6-fold (4 days) in comparison with BSA alone (Figure 1C,D).

The fluorescence intensities relative to AGEs in the incubations of BSA in the presence of CvExt, but in the absence of glycative agents (glucose or MG), were significantly low and similar to those values observed in the incubations of BSA alone throughout the monitoring period (Figure 1A,C). These findings suggested that CvExt does not contain metabolites able to emit fluorescence (when excited at 355 nm) at the same wavelength used to monitor the fluorescent AGEs (430 nm), leading us to believe that there are no interferences in the results of the fluorescent AGEs when CvExt was incubated with BSA + glucose or with BSA + MG.

In the BSA + glucose system, CvExt significantly decreased the formation of AGEs in a concentration-dependent manner throughout the incubation period. After 30 days, the values of fluorescent AGEs were decreased by 30%, 42%, and 70% in the incubations of BSA + glucose with 62.5, 125, and 250 µg/mL CvExt, respectively, in comparison with BSA + glucose (Figure 1B). It is important to mention that, over the 30 days of incubation, 125 µg/mL CvExt exhibited a profile similar to that of AG about its ability to inhibit the AGE formation, whereas 250 µg/mL CvExt was even more effective than AG in promoting a decrease in the AGE levels. The levels of fluorescent AGEs in samples having BSA + glucose + 250 µg/mL CvExt were similar from those of BSA alone, which allow us to suggest that *C. verticillata* extract is able to prevent AGE formation in vitro (Figure 1B).

In the BSA + MG system, the CvExt decreased the AGE levels in a concentration-dependent manner, at concentrations of 250 µg/mL (1, 2 and 4 days), 125 µg/mL (1 and 2 days), and 62.5 µg/mL (1 day). After 4 days, the levels of fluorescent AGEs were decreased by 32% in the incubations having BSA + MG + 250 µg/mL CvExt, in comparison with BSA + MG, while CvExt at concentrations of 62.5 and 125 µg/mL was not able to inhibit the AGE formation in BSA + MG incubations (Figure 1D).

The absorption spectra of incubation samples containing BSA + CvExt (Figure S3A,B), BSA + glucose + CvExt after 30 days (Figure S4A), and BSA + MG + CvExt after 4 days (Figure S4B) were monitored. Incubations with CvExt had low absorption capacities between 300 and 400 nm, an interval that encompasses the wavelength used to excite the fluorescent AGEs (355 nm) (Figure S3A,B). An intriguing data were observed in the absorption spectrum from BSA + MG + CvExt; between 300 and 400 nm, incubations samples containing BSA + MG + CvExt exhibited higher absorbance values than those of BSA + MG (Figure S4B); however, the same occurred in incubations having BSA + MG + AG (Figure S4B). It can be suggested that reactions between MG and AG or MG and CvExt metabolites generate products that have absorption capacity between 300 and 400 nm, which can act competitively with the AGEs monitored in the present study. Therefore, the minor fluorescence intensities relative to AGEs observed in incubations of BSA + MG + CvExt (especially at 250 µg/mL) must be interpreted with caution, taking into account the possibility of this analytical interference. However, this possible interference is inapplicable to CvExt when incubated with BSA + glucose. After 30 days of incubation, the absorbance values of BSA + glucose + CvExt were similar to those of BSA + glucose (Figure S4A), and thus, the significant anti-AGE activity of CvExt in the BSA + glucose system must indeed be an important biological activity and not an analytical interference of *C. verticillata* metabolites.

**Figure 2:**
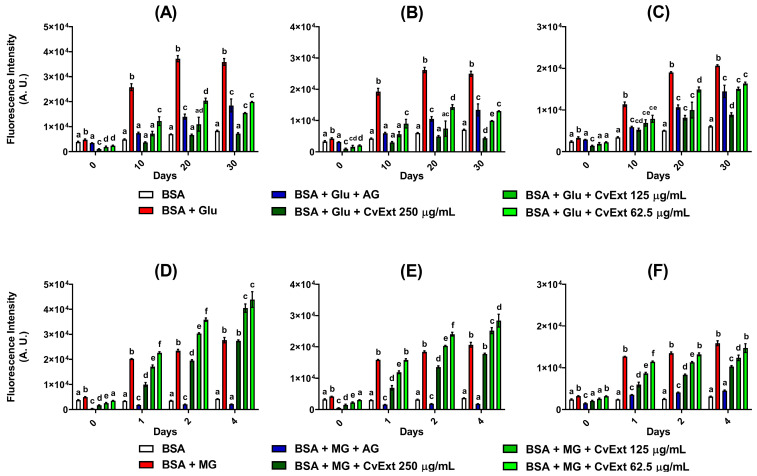
Effects of CvExt on the formation of dityrosine (**A**,**D**), *N*′-formylkynurenine (**B**,**E**), and kynurenine (**C**,**F**) using in vitro model systems of protein glycation based on BSA + glucose or BSA + MG. (**A**) Dityrosine levels in BSA + glucose; (**B**) *N*′-formylkynurenine in BSA + glucose; (**C**) kynurenine levels in BSA + glucose; (**D**) dityrosine levels in BSA + MG; (**E**) *N*′-formylkynurenine in BSA + MG; (**F**) kynurenine levels in BSA + MG. CvExt—*Cissus verticillata* leaf hydroethanolic extract; BSA—bovine serum albumin; Glu—glucose; MG—methylglyoxal; AG—aminoguanidine. Values are expressed as the mean ± SEM. Differences between groups were considered significant if *p* < 0.05 and were analyzed using one-way ANOVA followed by the Student−Newman−Keuls test. Means not sharing a common letter indicate significant differences.

The fluorescence intensities of dityrosine, *N*′-formylkynurenine, and kynurenine were monitored, representing the oxidative changes of tyrosine and tryptophan amino acid residues. After 30 days of incubation with BSA + glucose, the fluorescence relative to dityrosine, *N*′-formylkynurenine, and kynurenine were increased 4.3-fold, 3.5-fold, and 3.4-fold, respectively (Figure 2A–C). For BSA + MG incubated for 4 days, the fluorescence signals relative to dityrosine, *N*′-formylkynurenine, and kynurenine were increased 6.5-fold, 5.6-fold, and 5.1-fold, respectively (Figure 2D–F).

The best effects of *C. verticillata* extract on decreasing the biomarkers of tyrosine and tryptophan oxidation were achieved in incubations with BSA + glucose. In BSA + glucose, CvExt strongly inhibited the formation of dityrosine, *N*′-formylkynurenine, and kynurenine in a concentration-dependent manner, reaching after 30 days values that were lower (250 µg/mL CvExt) or similar (125 and 62.5 µg/mL CvExt) than the corresponding values of BSA + glucose + AG (Figure 2A–C).

On the other hand, incubation of BSA + MG with CvExt for 4 days resulted in marginal decreases in the levels of kynurenine at 125 and 250 µg/mL of CvExt (Figure 2F). Furthermore, after 4 days of incubation with BSA + MG, CvExt was not able to reduce the levels of dityrosine (Figure 2D) or *N*′-formylkynurenine (Figure 2E); on the contrary, the fluorescence signals relative to dityrosine and *N*′-formylkynurenine were significantly increased in incubations having BSA + MG + CvExt at concentrations of 62.5 and 125 µg/mL.

**Figure 3:**
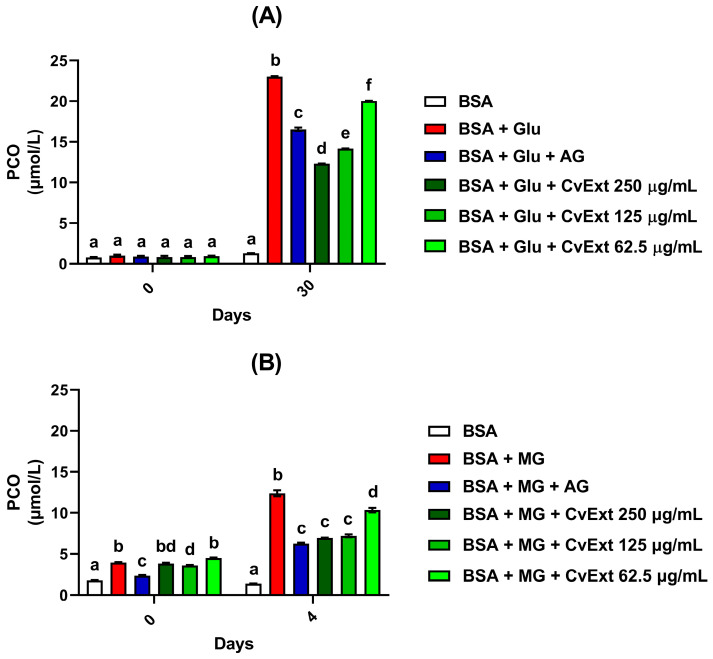
Effects of CvExt on PCO levels using in vitro model systems of protein glycation based on BSA + glucose or BSA + MG. (**A**) PCO levels in BSA + glucose after 30 days; (**B**) PCO levels in BSA + MG after 4 days. CvExt—*Cissus verticillata* leaf hydroethanolic extract; BSA—bovine serum albumin; Glu—glucose; MG—methylglyoxal; AG—aminoguanidine; PCO—protein carbonyl groups. Values are expressed as the mean ± SEM. Differences between groups were considered significant if *p* < 0.05 and were analyzed using one-way ANOVA followed by the Student−Newman−Keuls test. Means not sharing a common letter indicate significant differences.

In addition to decreasing the in vitro formation of products related to the oxidation of tyrosine and tryptophan residues, CvExt also reduced the PCO levels when incubated with BSA + glucose or BSA + MG. In incubations of BSA + glucose, the PCO levels were decreased by 13%, 38%, and 47% in the presence of 62.5, 125, and 250 µg/mL CvExt, respectively (Figure 3A), while in BSA + MG, the PCO levels were decreased by 17%, 42%, and 44% in the presence of 62.5, 125, and 250 µg/mL CvExt, respectively (Figure 3B).

### 2.3. LC-MS/MS Chemical Profile of CvExt and Its Fractions

**Figure 4:**
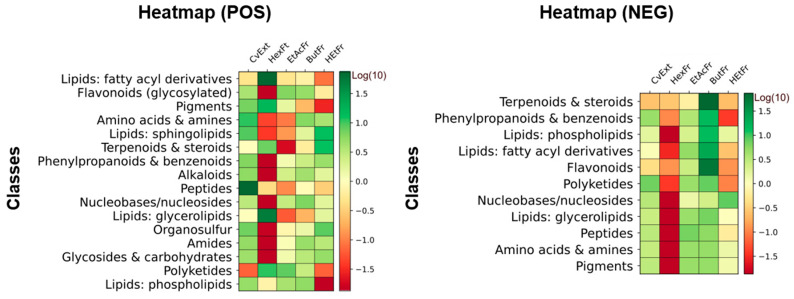
Heatmap representation of metabolite class distribution across solvent fractions of the *Cissus verticillata* extract. The color intensity reflects the scaled relative contribution of each metabolite class, allowing a comparison of class-level compositional differences among fractions. LC–MS/MS peak intensities (ESI^+^ and ESI^−^) were log_10_-transformed and normalized to relative abundance prior to visualization. CvExt—*Cissus verticillata* leaf hydroethanolic extract; HexFr—hexane fraction of CvExt; EtAcFr—ethyl acetate fraction of CvExt; ButFr—butanolic fraction of CvExt; HEtFr—hydroethanolic fraction fraction of CvExt.

The LC–MS/MS analysis in both positive and negative ionization modes revealed a chemically diverse profile. In positive mode, the dataset was dominated by amino acids and their derivatives, including canonical amino acids (L-arginine, L-histidine, L-phenylalanine, and L-tryptophan) and small peptides (Figure 4). A considerable number of O-glycosylated flavonoids were also detected, such as rutin, quercitrin, kaempferol rhamnosides, myricetin glycosides, and cyanidin-3-O-rutinoside, highlighting the abundance of flavonoid conjugates (Figure 4). Lipid-related metabolites were another major group, encompassing free fatty acids, fatty acid conjugates, glycerolipids, glycerophospholipids, fatty alcohols, and amides (Figure 4). Chlorophyll catabolites, particularly pheophorbide A and hydroxypheophorbide derivatives, were consistently observed, together with carotenoids (Figure 4). Additional classes included phenolic acids and cinnamic acid derivatives (e.g., chlorogenic acids, coumaric acids), terpenoids and triterpenes (oleanolic acid, echinocystic acid, and kaurenol), and several alkaloids (indole, pyridine, and purine) (Figure 4).

The negative ionization mode confirmed many of these classes, particularly phenolic acids, cinnamic acid derivatives, and O-glycosylated flavonoids, and further highlighted triterpenes, saponins, and lipid species (Figure 4). Together, these results indicate that the metabolome is enriched in primary metabolites (amino acids, fatty acids) as well as secondary metabolites of high structural diversity, especially flavonoids, phenolic acids, terpenoids, and chlorophyll derivatives (Figure 4).

**Figure 5:**
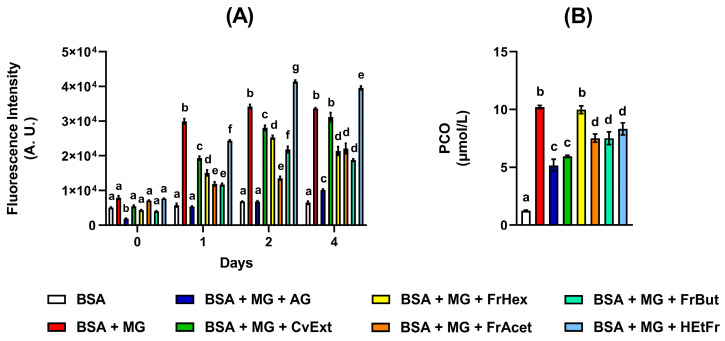
Effects of 125 µg/mL of CvExt, HexFr, EtAcFr, ButFr, and HEtFr on the formation of fluorescent AGEs using in vitro model systems of protein glycation based on BSA + MG (**A**) and PCO levels in BSA + MG after 4 days (**B**). CvExt—*Cissus verticillata* leaf hydroethanolic extract; HexFr—hexane fraction of CvExt; EtAcFr—ethyl acetate fraction of CvExt; ButFr—butanolic fraction of CvExt; HEtFr—hydroethanolic fraction fraction of CvExt; BSA—bovine serum albumin; MG—methylglyoxal; AG—aminoguanidine. Values are expressed as the mean ± SEM. Differences between groups were considered significant if *p* < 0.05 and were analyzed using one-way ANOVA followed by the Student−Newman−Keuls test. Means not sharing a common letter indicate significant differences.

**Figure 6:**
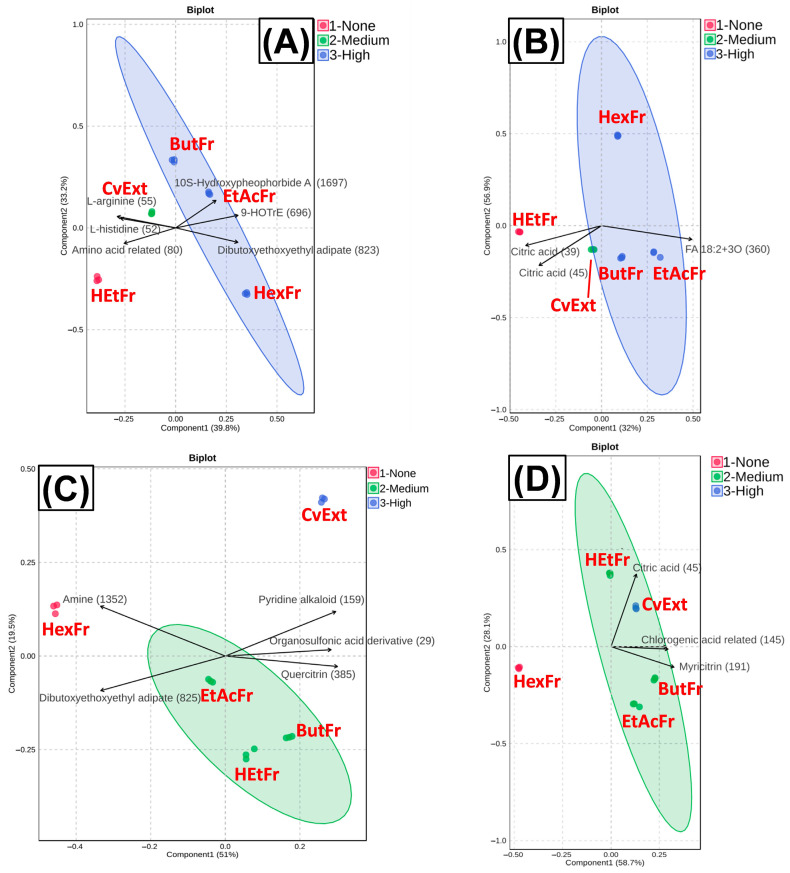
PLS-DA biplots based on LC-MS features in positive and negative ionization modes, illustrating the separation of extract and solvent fractions according to their anti-AGE and anti-PCO activities. (**A**) anti-AGE activity (positive ionization); (**B**) anti-AGE activity (negative ionization); (**C**) anti-PCO activity (positive ionization); (**D**) anti-PCO activity (negative ionization). CvExt—*Cissus verticillata* leaf hydroethanolic extract; HexFr—hexane fraction of CvExt; EtAcFr—ethyl acetate fraction of CvExt; ButFr—butanolic fraction of CvExt; HEtFr—hydroethanolic fraction fraction of CvExt. Numbers with brackets correspond to the MZmine ID of the annotated metabolites from *C*. *verticillata*, which can be found in Tables S1 and S2.

To better understand the relationship between metabolite composition and bioactivity, BSA + MG incubations with 125 µg/mL of CvExt, HexFr, EtAcFr, ButFr, or HEtFr were conducted in order to determine their respective anti-AGE and anti-PCO activities (Figure 5). After that, PLS-DA was applied to data from both ionization modes for results found with anti-AGE and anti-PCO assays (Figure 6).

In the anti-AGE dataset (Figure 6A,B), the HexFr clustered separately from the ButFr and EtAcFr, despite all showing inhibitory activity, while the crude extract lost effectiveness compared to the fractions. This suggests that distinct metabolite groups are responsible for anti-AGE activity in each fraction. In contrast, the anti-PCO assay (Figure 6C,D) revealed a different pattern: the crude extract exhibited higher activity than the individual fractions, while the HexFr showed no relevant effect, and the EtAcFr, ButFr and HEtFr clustered equidistantly from the extract. This indicates that, unlike in anti-AGE, the synergistic combination of metabolites in the extract enhanced the overall bioactivity related to the anti-PCO effect.

#### 2.3.1. Correlation of Annotated Metabolites with Anti-AGE Activity

The LC-MS/MS profiling of *Cissus* extracts in both ionization modes revealed complementary insights into the chemical drivers of the observed anti-AGE activity. Given that the PLS-DA plots indicated distinct metabolic profiles among these fractions, it is likely that different sets of metabolites drive the observed activities. For this reason, the correlation analysis presented below focuses specifically on the fractions with the highest anti-AGE activity, highlighting the annotated compounds most strongly associated with their bioactivity (Table 2).

Among the phenolic constituents detected, flavonoids represented the most recurrent group correlating with anti-AGE activity, particularly in the ButFr and EtAcFr fractions. Flavonoids such as rutin, quercitrin, vicenin-2, and kaempferol derivatives consistently exhibited strong positive correlations (Table 2). It is important to note that, despite their abundance in HEtFr, the lack of bioactivity in this fraction suggests that either the flavonoids’ concentration was insufficient or synergistic interactions with other compound classes (present in EtAcFr and ButFr, but not HEtFr) were necessary to elicit measurable activity.

Cinnamic acid derivatives also showed notable correlations. *p*-Coumaric acid, 3-*p*-coumaroylquinic acid, and related conjugates presented positive correlations across EtAcFr and ButFr, suggesting that hydroxycinnamates may complement flavonoid action (Table 2). Coumarins also emerged as correlated markers. 6,7-Dihydroxycoumarin and 7-(geranyloxy)coumarin exhibited positive associations with activity, particularly in EtAcFr and ButFr (Table 2).

In contrast to the more polar EtAcFr and ButFr, the anti-AGE activity observed in HexFr was predominantly associated with lipophilic metabolites, notably unsaturated fatty acids (e.g., oleic acid), glycerolipids, pentacyclic triterpenoids (oleanane/ursane types), and pheophorbide-related chlorophyll-like products (Table 2).

#### 2.3.2. Correlation of Annotated Metabolites with Anti-PCO Activity

In contrast to the anti-AGE assay, where fractionation improved activity, the anti-PCO results revealed that the extract retained the highest inhibitory effect, suggesting that the complete chemical mixture exerts additive or synergistic protection (Figure 6). However, the medium-activity fractions (EtAcFr, ButFr, and HEtFr) emerged as particularly relevant for identifying key contributors to this effect. To clarify which compounds underpin this intermediate activity, a correlation analysis was performed (Table 3).

The analysis highlighted the consistent involvement of several flavonoids, especially quercitrin, myricetin derivatives, and kaempferol *O*-rhamnoside, as strong correlates of activity across the three fractions. Complementarily, the presence of multiple cinnamic acid derivatives, including chlorogenic acid, neochlorogenic acid, p-coumaric acid, and coumaroyl-glucosides, aligns with the antioxidant potential of hydroxycinnamates (Table 3).

Coumarins and simple phenolic acids such as gentisic and kinic acid also contributed positively, reinforcing the role of small, polar phenolics. Additionally, amino acids such as tryptophan, phenylalanine, and homoarginine were associated with activity. The detection of triterpenoid signals, including kaji-ichigoside F1, indicates that non-phenolic metabolites may also modulate activity (Table 3).

The PLS-DA clustering indicated that the EtAcFr, ButFr, and HEtFr, while chemically distinct, share overlapping pools of active metabolites, explaining their intermediate yet significant performance. Together, these results suggest that anti-PCO activity in *C. verticillata* relies on the concerted action of multiple phenolic subclasses, with the crude extract maintaining the optimal balance, while medium-activity fractions illuminate the specific compounds driving the effect (Figure 6 and Table 3).

## 3. Discussion

The onset of oxidative stress is characterized by overproduction of ROS and/or failure in the intracellular antioxidant defenses, which contributes to the pathogenesis of several diseases including DM [17]. From the findings of the present study, it was observed that CvExt exhibited low scavenging capacity towards ROO^•^ in comparison with standard antioxidants (Figure S1, Table 1), which is partially expected considering that isolated substances (quercetin and Trolox) frequently exhibit superior antioxidant potentials when compared to plant preparations in terms of complex metabolite composition. In Lage et al. [18], the antioxidant activities of different tea extracts were determined by using the crocin bleaching assay for radical capture under lipophillic conditions and compared with standard antioxidants, including BHT (butyl-hydroxytoluene) and Trolox. The study showed that the antioxidant activities of the tea extracts observed in concentrations of g/L (powder material extracted) were equivalent to the responses obtained for BHT and Trolox at much lower concentrations, as μM or nM, which is in agreement with our findings.

In the O_2_^•−^ scavenging assay, CvExt showed an in vitro antioxidant activity lower than quercetin, bur similar to Trolox (Figure S2, Table 1), indicating a promising antioxidant activity of *C. verticillata* leaf extract. By evaluating the ability of the aqueous extract of *C. verticillata* leaves to capture O_2_^•−^ under experimental conditions relatively similar to those used in the present study, Khalil et al. [19] found an EC_50_ value = 60.0 ± 2.3 μg/mL, which was about 6.5 times lower than the value found in our study for CvExt (EC_50_ = 346.08 ± 11.38 μg/mL) (Table 1), a discrepancy that can be attributed to qualitative and quantitative differences in the chemical composition of the plant extracts, and to different techniques and solvents used to prepare the extracts.

**Figure 7:**
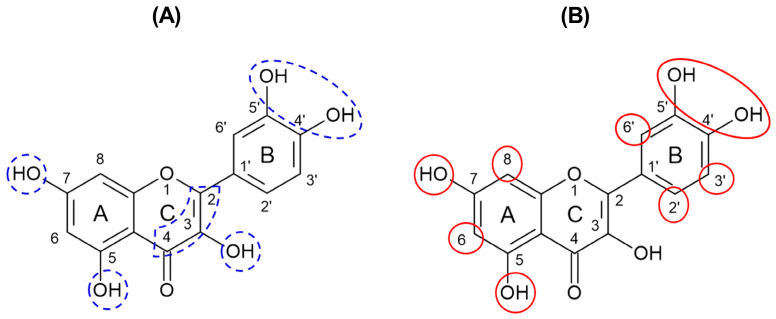
Common structure of flavonoids with potential sites responsible for antioxidant (**A**) and antiglycation (**B**) activities. Based on Crascì et al. [21] and Li et al. [28]. According to the cited literature, the sites circled in blue are important for the antioxidant activity; the sites circled in red are important for the antiglycation activity.

Considering the main active metabolites presented in CvExt and identified by LC-MS/MS analysis (Table 2 and Table 3), it is reasonable to suggest that polyphenols are responsible for the ROS scavenging potential demonstrated by CvExt. The polyunsaturated structures of polyphenols can stabilize the unpaired electrons of radical species by incorporating them into more energetically favorable resonance structures [20,21,22]. Flavonoids and their derivatives deserve special attention. They have well-documented antioxidant activities related to the ortho-dihydroxy (catechol) structures in the B ring and the hydroxyl group at position 7 in the A ring (Figure 7A), which confer high stability to the flavonoid phenoxyl radicals via hydrogen bonding or by expanded electron delocalization, and a C2−C3 double bond in conjugation with a 4-oxo function in the C ring (Figure 7A) responsible for determining the co-planarity of the heteroring, participating in radical stabilization via electron delocalization across all three ring systems. In addition, the hydroxyl groups at positions 3 (C ring) and 5 (A ring) (Figure 7A) improve the radical scavenging effect, providing a hydrogen bonding to the oxo-group [21,23].

Furthermore, flavonoids and their derivatives also have antiglycation activities [24,25,26,27], which can explain the ability of CvExt, EtAcFr, ButFr, and HEtFr to mitigate glycoxidative damages generated on the BSA structure when incubated with glucose or MG. The antiglycation properties of the flavonoids may be explained by the following: (i) the hydroxylation on the A and B rings (Figure 7B) contributes to the ability of flavonoids to inhibit AGE generation, which may be increased with the addition of hydroxyl groups at the positions 4′-, 5′-, 5-, and 7; (ii) the unsubstituted carbons C-2′, C-3′, C-6′, C-6, and C-8 (Figure 7B) contribute to the trapping of dicarbonyl species, mainly MG [21,28].

Among the flavonoids found in this study (Table 2 and Table 3), it can be highlighted the quercetin derivatives myricetin and kaempferol. Li et al. [28] found that these flavonoids were able to inhibit the generation of different AGEs, including N^ε^-carboxymethyl-lysine (CML), N^ε^-carboxyethyl-lysine (CEL), pyrraline, pentosidine, and argpyrimidine, in an in vitro model system of glycoxidative stress based on lysine and arginine incubated with glucose. Using an in vitro model system of protein glycation by glucose, Dias et al. [29] observed that rutin has anti-AGE and anti-PCO activities. Finally, in another study, the flavonoid vicenin-2 decreased the formation of fluorescent AGEs and PCO when incubated with BSA, fructose or glucose for 4 weeks, showing a better antiglycation potential than AG [30].

Cinnamic acid derivatives (Table 2 and Table 3) belong to another class of metabolites found in CvExt, EtAcFr, ButFr, and HEtFr that showed the ability to protect against in vitro glycoxidative stress. Previously, cinnamic acid derivatives were shown to decrease the generation of fluorescent AGEs and PCO content when studied in an in vitro model system of protein glycation with BSA incubated with fructose [31]. According to Qais et al. [32], the antiglycation potential of cinnamic acid may be associated with its ability to scavenge reactive dicarbonyl species (e.g., glyoxal and MG) and to form complexes with the albumin structure, stabilized mainly through hydrophobic and hydrogen bonds, thus promoting a steric blocking in one of the main glycating sites of this protein.

Similarly, coumarins are metabolites that also played an important role in the anti-AGE and, especially, in the anti-PCO activities exhibited by CvExt, EtAcFr, ButFr, and HEtFr (Table 2 and Table 3). The antiglication activity of coumarins may be related to the capacity of these molecules to scavenge reactive dicarbonyl species and to develop complexes with the albumin structure, which are stabilized by hydrophobic interactions and van der Waals forces, preventing the Maillard reaction [33].

Free amino acids found in EtAcFr and ButFr also contributed to the antiglycation activity of *C. verticillata* (Table 2 and Table 3), which can be associated to their ability to scavenge reactive dicarbonyl species [34,35]. According to Sulochana et al. [36], an approximately 11-fold decrease in the glycation rate of actin molecules was observed when the commercial actin standard was incubated with glucose and the amino acid arginine.

Lipophilic metabolites found in CvExt and its fractions (mostly in HexFr) exhibited significant anti-AGE activity (Table 2). The antiglycation activity associated to the unsaturated fatty acids is in line with the findings of Abdnim et al. [37]; the authors observed that the oil from the fruits of *Opuntia ficus-indica*, rich in fatty acids was able to inhibit the generation of AGEs after incubation with BSA and glucose. Furthermore, the identified pheophorbide-related chlorophyll-like compounds (Table 2) also had the ability to inhibit protein glycation, as previously reported. Hong et al. [38] observed that pheophorbide A was able to reduce the formation of CML when incubated with BSA and glycolaldehyde. In addition, the anti-AGE activity of the pentacyclic triterpenoids (Table 2) reinforces previous findings; compounds belonging to this metabolite class (e.g., astragalosides, boswellic acid, corosolic acid) attenuated the formation of AGEs and their precursors in in vitro model systems of protein glycation [39].

## 4. Materials and Methods

### 4.1. Reagents and Chemicals

The following reagents were used: 2,2′-azobis(2-amidinopropane) dihydrochloride (AAPH, Sigma-Aldrich, St. Louis, MO, USA), 2,4-dinitrophenylhydrazine (DNPH, Sigma-Aldrich, USA), 6-hydroxy-2,5,7,8-tetramethylchroman-2-carboxylic acid (Trolox, Sigma-Aldrich, USA), acetonitrile (Sigma-Aldrich, USA), aminoguanidine (AG, Sigma-Aldrich, USA), anhydrous bibasic sodium phosphate (Labsynth, Diadema, SP, Brazil), bovine serum albumin (BSA, Sigma-Aldrich, USA), butanol (Labsynth, Brazil), crocin (Sigma-Aldrich, USA), dimethyl sulfoxide (Sigma-Aldrich, USA), ethanol (Labsynth, Brazil), ethyl acetate (LS Chemicals, Mumbai, Maharashtra, India), formic acid (Sigma-Aldrich, USA), glucose (Sigma-Aldrich, USA), hexane (Sigma-Aldrich, USA), methylglyoxal (MG, Sigma-Aldrich, USA), monobasic sodium phosphate monohydrate (Labsynth, Brazil), nicotinamide adenine dinucleotide (NADH, Sigma-Aldrich, USA), nitrotetrazolium blue (NBT, Sigma-Aldrich, USA), phenazine metasulfate (PMS, Sigma-Aldrich, USA), quercetin (Sigma-Aldrich, USA), and sodium azide (Merck, Darmstadt, Hesse, Germany).

### 4.2. Plant Material, Extraction, and Fractionation

Leaves of *Cissus verticillata* (L.) Nicolson & C.E. Jarvis (syn. *Cissus sicyoides* L.) were collected at the Garden of Medicinal Plants of the School of Pharmaceutical Sciences of São Paulo State University (UNESP), Araraquara Campus, São Paulo, Brazil (21°48′58.1″ S, 48°12′04.2″ W) on 26 October 2020 at 12:00 a.m. (30 °C, 60% relative humidity, sunny weather). This research is registered at the National System for the Management of Genetic Heritage and Associated Traditional Knowledge (SisGen; Register A109280). A voucher specimen was deposited at the Herbarium of Ilha Solteira (HISA, Ilha Solteira, São Paulo, Brazil) under the number 10936.

The leaves were dried in an oven (40 °C) with air circulation for 5 days and then powdered in a knife mill. Dried and milled leaves (133.08 g) were submitted to extraction with an ethanol/water ratio of 70:30 (*v*/*v*) by remaceration in 2 steps at 30 ± 5 °C under occasional stirring. The extraction was performed for 72 h in each step. The total plant drug/solvent ratio was 1:5 (1 kg/5 L). The ethanol was removed by rotary evaporation and the water was removed by freeze-drying, which ensured the production of a solvent-free powder of the *C*. *verticillata* leaf hydroethanolic extract (CvExt) yielding 17.00 g, which was stored at −20 °C and protected from light and humidity until the moment of the use.

To optimize the process of identifying the chemical composition of the plant extract obtained, 1.0035 g of CvExt was subjected to sequential liquid−liquid extractions with hexane, ethyl acetate, butanol. and an ethanol/water ratio of 70:30 (*v*/*v*). After removal of organic solvents by rotary evaporation and water by freeze-drying, the following fractions were obtained: hexane fraction (HexFr; yielding 0.0331 g), ethyl acetate fraction (EtAcFr; yielding 0.0369 g), butanolic fraction (ButFr; yielding 0.1562 g), and hydroethanolic fraction (HEtFr; yielding 0.4811 g). All plant preparations were stored at −20 °C until the moment of use in the experiments and analysis.

### 4.3. In Vitro Antioxidant Assays

To determine the in vitro antioxidant activity of CvExt, scavenging assays towards O_2_^•−^ and ROO^•^ were performed. Quercetin and Trolox (soluble synthetic analogue of vitamin E) were used as standard antioxidants since they have well-established antioxidant activities [40].

#### 4.3.1. ROO^•^ Scavenging Assay

The ROO^•^ is formed during the process of lipoperoxidation in an aerated medium. The crocin bleaching assay simulate this event by the decrease in its absorbance at 443 nm induced by the addition of the azo-compound AAPH, which by thermolysis at 40 °C generates ROO^•^ at a constant rate. Antioxidants compete with the crocin for ROO^•^, where the inhibition of its oxidation represents the capacity of samples to capture the radical species [41,42].

The assay was performed in triplicate, according to Assis et al. [41] with modifications. Crocin was mixed with various concentrations of the samples in sodium phosphate buffer (120 mmol/L, pH 7.0). The reaction was started by adding 12.5 mmol/L AAPH and performed at 40 °C with constant stirring. The rate of the crocin bleaching (linear after about 1 min of reaction) was monitored at 443 nm for 9 min. Possible interferences of the samples were eliminated by preparing a reaction mixture without crocin for each sample and used as the reaction blank.

The decrease in the crocin bleaching rate in the presence of an antioxidant was analyzed by the kinetic equation of competition:

v_0_/v = 1 + slope · [S]/[C]

where v_0_ is the velocity in the absence of a compound; v is the velocity in the presence of a compound; [S] is the sample (CyExt or Quercetin or Trolox) concentration; [C] is the crocin concentration (25 µmol/L). The slope of the regression line indicates the relative capacity of a compound to interact with ROO^•^.

#### 4.3.2. O_2_^•−^ Scavenging Assay

The interaction between NADH, PMS, and molecular oxygen (O_2_) results in the generation of O_2_^•−^ [43]. The O_2_^•−^ is capable to reduce the NBT to a formazan, which exhibits a blue color directly proportional to the radical concentration. The assay was performed in triplicate, where PMS (372 μmol/L), NBT (600 μmol/L), NADH (1560 μmol/L), and various concentrations of the CvExt or standards were added in sodium pyrophosphate buffer (25 mmol/L, pH 8.3). After 7 min in the dark at room temperature, the absorbance was monitored at 560 nm to determine the concentration of formazan [41,44]. The assay in the absence of the CvExt or standards was used as a control (100% reaction) and the reaction medium without NADH was used as a reading blank. The results were expressed as mean ± SEM of the 50% effective concentration (EC_50_).

### 4.4. In Vitro Model Systems of Protein Glycation

The in vitro model system of protein glycation was performed with incubations of BSA and glucose, and was performed according to Dos Santos et al. [45] and Dias et al. [29]. BSA (10 mg/mL) was incubated with glucose (0.5 M) in phosphate buffer (0.1 M, pH 7.4) containing 0.02% sodium azide at 37 °C for 30 days.

Another in vitro model system of protein glycation was performed with incubations of BSA with MG and was performed according to Rodrigues et al. [46]. BSA (10 mg/mL) was incubated with MG (2 mM) in phosphate buffer (0.1 M, pH 7.4) containing 0.02% sodium azide at 37 °C for 4 days.

All incubation conditions were performed in triplicate. BSA (without glucose or MG), BSA + glucose, and BSA + MG were incubated in the absence or presence of AG (1 mM), a prototype therapeutic agent with anti-AGE activity, and also in the absence or presence of CvExt (62.5, 125, and 250 μg/mL) as well as its fractions (125 μg/mL). Dimethyl sulfoxide (5% *v*/*v*) was used as a cosolvent in the solubilization of CvExt, HexFr, EtAcFr, ButFr, and HEtFr.

Aliquots from the incubations were collected at 0, 10, 20, and 30 days (for the in vitro model system of protein glycation with BSA + glucose) or at 0, 1, 2, and 4 days (for the in vitro model system of protein glycation with BSA + MG) to measure the generation of fluorescent AGEs and markers of amino acid oxidation, and levels of protein carbonyl groups.

#### 4.4.1. Fluorescent AGEs and Markers of Amino Acid Oxidation

Fluorescent AGEs and markers of amino acid oxidation were monitored spectrofluorometrically, at the respective excitation/emission wavelengths: AGEs (355/430 nm) [47,48]; dityrosine (330/415 nm) as a marker of tyrosine oxidation; *N*′-formylkynurenine (325/434 nm) and kynurenine (365/480 nm) as markers of tryptophan oxidation [49]. The fluorescence intensities of these markers were measured using a multimode microplate reader, with a split set at 16 nm (Synergy TM H1, BioTek Instruments Inc., Winooski, VT, USA).

The fluorescence values of AGEs, dityrosine, kynurenine, and *N*′-formylkynurenine were obtained after the arithmetic subtraction of the fluorescence from incubations of AG, CvExt, or fractions with buffer (without BSA) from those of AG or CvExt or fractions incubated with BSA, BSA + glucose, or BSA + MG. The results were expressed in terms of arbitrary units (A. U.) of fluorescence.

#### 4.4.2. Protein Carbonyl Groups

The levels of protein carbonyl groups (PCO) were measured as described by Levine et al. [50] and Meeprom et al. [48], with modifications. PCO reacts with DNPH, generating dinitrophenylhydrazone, which was monitored at 370 nm. PCO levels were estimated using the molar extinction coefficient of the hydrazone (22.000 M^−1^.cm^−1^) and the results are expressed in terms of μmol/L.

### 4.5. LC-MS/MS Chemical Profiling of CvExt

CvExt and its fractions’ chemical profiling was performed using a Waters ACQUITY I-Class UPLC (ultra-performance liquid chromatographer) (Waters®, Milford, MA, USA) system coupled to a Waters Xevo G2-XS high-resolution quadrupole time-of-flight (QTOF) mass spectrometer equipped with an electrospray ionization (ESI) source operating in both positive and negative ion modes. Chromatographic separation was achieved on a Waters C18 Acquity UPLC^®^HSS T3 column (1.8 μm; 2.1 mm × 100 mm) maintained at 35 °C. The mobile phases consisted of solvent A (water with 0.1% formic acid) and solvent B (acetonitrile with 0.1% formic acid). The following gradient was applied: 0–8 min: 5–100% B; 8–14 min: 100% B. The flow rate was set at 0.35 mL/min, and the injection volume was 1 uL.

Data-dependent acquisition (DDA) was employed over *m*/*z* 100–1500. Source parameters were as follows: capillary voltage, 2.5 kV; cone voltage, 20 V; source temperature, 120 °C. Nitrogen served as nebulizing and desolvation gas; argon was used as the collision gas. Post-acquisition lockmass correction was applied using leucine enkephalin (pos: *m*/*z* 556.2771 [M + H]^+^, neg: *m*/*z* 554.2615 [M − H]^−^).

Raw LC–MS/MS data were converted to mzML format [51] and processed using MZmine [4.5.0] [52] for peak detection, deconvolution, and feature alignment. Processed data were exported in .mgf format and submitted to the GNPS Molecular Networking platform (Global Natural Products Social Molecular Networking, https://gnps.ucsd.edu, acessed on 30 August 2025) for spectral similarity searches against public MS/MS reference libraries. Putative annotations were obtained when experimental spectra showed high cosine similarity scores with reference entries. In parallel, in silico structure prediction and class assignment were performed using SIRIUS [6.0.7] [53] coupled with CSI:FingerID [54], CANOPUS [55], ClassyFire [56], and NPClassifier [57]. This allowed the assignment of molecular formulas, fragmentation tree-based ranking of candidate structures, and probabilistic classification into chemical classes.

Additionally, an in-house library was curated from compounds previously reported in the genus *Cissus* (Vitaceae) using the NuBBE Database (NuBBEDB) [58] and LOTUS natural products repository [59]. Reported metabolites were compiled and used as a taxonomically informed reference list for annotation. Candidate features showing agreement in precursor ion *m*/*z*, fragmentation patterns, and taxonomic plausibility were marked as tentative annotations. Further details of LC-MS/MS data processing can be found in Supplementary Materials (Tables S1–S8).

### 4.6. Statistical Analysis

Results were expressed as means ± standard error of the mean (SEM) and were analyzed using one-way Analysis of Variance (ANOVA) followed by analysis of differences by the Newman−Keuls test. The software used was GraphPad Prism 9 (GraphPad Software, Boston, MA, USA). The level of statistical significance considered was *p* < 0.05.

Raw LC-MS peak intensity data were log10-transformed. For PLS-DA, Pareto scaling was applied, whereas Spearman’s rank correlations were performed on log10-transformed data only. A fifth of the lowest positive number was imputed to blank data. An IQR filter was applied (10% for positive spectra data; 25% for negative spectra data). Bioactivity values were normalized to a 0–1 range and classified into three categories using 33% percentile thresholds (low, medium, and high activity). All analyses were conducted in MetaboAnalyst (https://www.metaboanalyst.ca/, acessed on 30 August 2025).

## 5. Conclusions

The hydroethanolic extract obtained from *C. verticillata* leaves exhibited scavenging capacity towards ROS, mainly O_2_^•^*^−^*, and had a significant ability to decrease in vitro the levels of fluorescent AGEs, markers of tyrosine and tryptophan oxidation, and protein carbonyl groups. By using LC-MS/MS and PLS-DA techniques, flavonoids, cinnamic acid derivatives, coumarins, free amino acids, and some lipophilic compounds were the main metabolites found in the *C. verticillata* that, individually or in combination, may be responsible for the ROS scavenging activity and the in vitro antiglycation potential of the plant preparations. The leaves of *C. verticillata* represent a promising source of compounds with the potential to inhibit glycoxidation processes, constituting a relevant target for investigation as a complementary therapeutic approach to diabetes-related complications.

## Figures and Tables

**Table 1:** Comparison between ROS scavenging activities (ROO^•^ and O_2_^•−^) of quercetin, Trolox, and CvExt (sample), expressed as the concentration needed for 50% inhibition (EC_50_ ± SEM, μg/mL) and the linear regression slope for the crocin bleaching assay.

Standards/Sample	O_2_^•− (^*^)^	ROO^• (^*^)^	Linear Regression Slope ^(^**^)^
Quercetin	25.93 ± 1.48 ^a^	1.11 ± 0.04 ^a^	24.796 ± 1.063 ^a^
Trolox	572.70 ± 99.95 ^b^	1.76 ± 0.12 ^a^	15.592 ± 1.294 ^b^
CvExt	346.08 ± 11.38 ^b^	29.98 ± 1.27 ^b^	0.972 ± 0.073 ^c^

^(^*^)^ EC_50_ ± SEM values obtained through the equation: %In = [1 − (ΔV/ΔV_0_)] · 100. ^(^**^)^ y = v_0_/v; x = [CvExt or antioxidant standards]/[C]. The values of angular coefficient ± SEM indicate the relative antioxidant capacity of the substance. CvExt—*Cissus verticillata* leaf hydroethanolic extract. Values are expressed as the mean ± SEM. Differences between groups were considered significant if *p* < 0.05 and were analyzed using one-way ANOVA followed by the Student−Newman−Keuls test. Means not sharing a common letter indicate significant differences.

**Table 2:** Representative annotated metabolites highly correlated with anti-AGE activity in the most active fractions of *Cissus verticillata*.

Compound (Class)	Ion Mode	HexFr	EtAcFr	ButFr
AMINO ACIDS				
L-phenylalanine (176)	POS	−0.48	−0.47	0.74
Val-Leu (227)	POS	−0.48	−0.47	0.95
Tryptophan (79)	NEG	−0.48	0.95	0.95
^(^*^)^ Amino acid-related (1346)	POS	0.96	0.17	0.00
ALKALOIDS				
1H-pyrrolo[3,2-b]pyridine-5-carboxylic acid (241)	POS	−0.48	−0.16	0.95
^(^*^)^ Alkaloid (258)	POS	−0.48	0.47	0.95
FLAVONOIDS				
Corymboside (282)	POS	−0.48	−0.05	0.95
Isoshaftoside (305)	POS	−0.48	−0.26	0.95
Vicenin 2 (262)	POS	−0.48	−0.47	0.95
Kaempferol O-rhamnoside (430)	POS	−0.48	0.95	0.74
Quercitrin (385)	POS	−0.47	0.95	0.95
Rutin (335)	POS	−0.33	0.95	0.95
Dihydrokaempferol (271)	NEG	0.00	0.96	0.48
Taxifolin (219)	NEG	0.00	0.91	0.85
CINNAMIC ACIDS				
3-*p*-coumaroylquinic acid (287)	POS	−0.48	0.53	0.95
*p*-coumaric acid (323)	POS	−0.48	0.58	0.95
2-({6-O-[(2E)-3-(4-Hydroxyphenyl)-2-propenoyl]-beta-D-glucopyranosyl}oxy)-3-phenylacrylic acid (277)	NEG	0.00	0.96	0.96
CHLOROPHYLL-LIKE				
10S-Hydroxypheophorbide A (1697)	POS	0.95	0.48	0.48
^(^*^)^ Pheophorbide A related (1666)	POS	0.96	0.59	0.48
FATTY COMPOUNDS				
12-hydroxyjasmonic acid-related (730)	POS	0.96	0.48	0.00
4-acetylbutyric-acid_1-octacosanol (1744)	POS	0.47	0.47	0.84
9-HOTrE (696)	POS	0.95	0.48	0.48
Tetradecanoic acid (496)	POS	−0.47	−0.47	0.95
^(^*^)^ Fatty amide (1328)	POS	0.33	0.54	0.96
4-oxododecanedioic acid (252)	NEG	0.44	0.96	0.96
FA 18:2 + 3O (360)	NEG	0.47	0.95	0.95
^(^*^)^ Unsaturated fatty acid (557)	POS	−0.47	−0.47	0.95
Oleic acid (1254)	POS	0.95	0.47	0.95
GLYCEROLIPIDS				
^(^*^)^ 1-Monolinoleoyl-rac-glycerol-related (2051)	POS	0.91	0.00	0.00
1-Oleoyl-sn-glycero-3-phosphoethanolamine related (734)	POS	0.17	0.48	0.96
Glc-Glc-octadecatrienoyl-sn-glycerol (441)	NEG	0.48	0.96	0.96
PI(18:1/0:0) (444)	NEG	0.00	0.48	0.96
^(^*^)^ Endocannabinoid (1372)	POS	0.96	0.48	0.00
CAROTENOIDS				
(2R)-beta,beta-Caroten-2-ol (699)	POS	0.87	0.29	0.00
COUMARINS				
6,7-Dihydroxycoumarin (139)	NEG	−0.48	0.95	0.95
7-(geranyloxy)coumarin (450)	NEG	0.00	0.17	0.96
TERPENES				
Kaji-ichigoside F1 (352)	NEG	0.00	0.96	0.96
Echinocystic acid (561)	NEG	0.96	0.96	0.00
Oleanolic acid (1323)	POS	0.96	0.33	0.00
TERPENES				
^(^*^)^ Triterpenoid (1860)	POS	0.96	0.64	0.48
PHENOLIC ACIDS				
Gentisate (114)	NEG	−0.48	0.95	0.95
Kinic acid (97)	NEG	−0.48	0.95	0.95

^(^*^)^ Class-level annotation. NEG—negative; POS—positive; HexFr—hexane fraction of CvExt; EtAcFr—ethyl acetate fraction of CvExt; ButFr—butanolic fraction of CvExt.

**Table 3:** Representative annotated metabolites highly correlated with anti-PCO activity in the most active fractions of *Cissus verticillata*.

Compound (Class)	Ion Mode	EtAcFr	ButFr	HEtFr
FLAVONOIDS				
Cyanidin-3-O-rutinoside (366)	POS	0.85	0.48	0.85
Kaempferol-O-rhamnoside (429)	POS	0.96	0.64	0.96
FLAVONOIDS				
Myricetin-3-Xyloside (344)	POS	0.96	0.48	0.96
Quercitrin (386)	POS	0.96	0.48	0.96
Dihydrokaempferol (271)	NEG	0.48	0.96	0.85
Myricetin-3-O-galactoside (164)	NEG	0.48	0.48	0.85
Apigenin-Hex-Hex (121)	NEG	0.54	0.48	0.96
Apigenin-Pen-Hex (148)	NEG	0.48	0.48	0.96
AMINO ACIDS				
L-arginine (55)	POS	0.48	0.96	0.48
L-histidine (52)	POS	0.48	0.96	0.48
L-phenylalanine (176)	POS	0.96	0.70	0.96
Val-Leu (227)	POS	0.96	0.48	0.96
Homoarginin (9)	NEG	0.96	0.96	0.48
Tryptophan (79)	NEG	0.48	0.48	0.96
Pantothenic acid-B5 (64)	NEG	0.48	0.48	0.96
ALKALOIDS				
1H-pyrrolo[3,2-b]pyridine-5-carboxylic acid (241)	POS	0.96	0.48	0.96
^(^*^)^ Alkaloid (258)	POS	0.96	0.48	0.96
CINNAMIC ACIDS				
3-*p*-coumaroylquinic acid (287)	POS	0.96	0.48	0.96
Neochlorogenic acid (239)	POS	0.96	0.59	0.96
*p*-coumaric acid (124)	POS	0.70	0.91	0.70
1-O-(4-Coumaroyl)-beta-D-glucose (104)	NEG	0.48	0.48	0.96
2-({6-O-[(2E)-3-(4-Hydroxyphenyl)-2-propenoyl]-beta-D-glucopyranosyl}oxy)-3-phenylacrylic acid (277)	NEG	0.48	0.48	0.85
3-Hydroxycinnamic acid (197)	NEG	0.48	0.48	0.87
Chlorogenic acid (133)	NEG	0.48	0.48	0.96
Coumaric acid (103)	NEG	0.48	0.48	0.96
Coumaroyl + C_6_H_9_O_8_ (90)	NEG	0.96	0.96	0.48
Coumaroyl + C_6_H_9_O_8_ (116)	NEG	0.96	0.96	0.59
CINNAMIC ACIDS				
Isochlorogenic acid B-related (94)	NEG	0.48	0.48	0.96
COUMARINS				
6,7-Dihydroxycoumarin (139)	NEG	0.48	0.48	0.96
7-(geranyloxy)coumarin (450)	NEG	0.87	0.48	0.85
PHENOLIC ACIDS				
Kinic acid (146)	NEG	0.48	0.48	0.96
Benzoic acid + 2O_O-Hex (63)	NEG	0.96	0.96	0.48
Gentisate (114)	NEG	0.48	0.48	0.96
TRITERPENES				
Kaji-ichigoside F1 (352)	NEG	0.48	0.48	0.85

^(^*^)^ Class-level annotation. Pen—undefined pentose moiety; Hex—undefined hexose moiety; NEG—negative; POS—positive; EtAcFr—ethyl acetate fraction of CvExt; ButFr—butanolic fraction of CvExt; HEtFr—hydroethanolic fraction of CvExt.

## Data Availability

The datasets generated during and/or analyzed during the current study are available from the corresponding author on reasonable request.

## Supplementary Materials

The following supporting information can be downloaded at: https://www.mdpi.com/article/10.3390/molecules31040697/s1, Table S1: Annotated metabolites from *Cissus verticillata* in positive mode (ESI^+^). Table S2: Annotated metabolites from *Cissus verticillata* in negative mode (ESI^−^). Table S3: Complete Spearman correlation analysis of anti-AGE activity in the most active fractions of *Cissus verticillata* leaf hydroethanolic extract (CvExt) in positive mode (ESI^+^). Table S4: Complete Spearman correlation analysis of anti-AGE activity in the most active fractions of fractions of *Cissus verticillata* leaf hydroethanolic extract (CvExt) in negative mode (ESI^−^). Table S5: Complete Spearman correlation analysis of anti-PCO activity in the most active fractions of *Cissus verticillata* leaf hydroethanolic extract (CvExt) in positive mode (ESI^+^). Table S6: Complete Spearman correlation analysis of anti-PCO activity in the most active fractions of *Cissus verticillata* leaf hydroethanolic extract (CvExt) in negative mode (ESI^−^). Table S7: LC-MS/MS processing parameters utilized on the MZMine software (v. 4.5.0). Table S8: Calculation parameters of SIRIUS for in silico annotation. Computations were made in positive and negative modes separately. Figure S1. Reaction velocity ratios plotted against the concentrations of the standard antioxidants or CvExt (sample) in the crocin bleaching assay (ROO^•^ scavenging assay): (A) quercetin; (B) Trolox; (C) CvExt. Figure S2: Capacity of the standard antioxidants or CvExt (sample) to scavenge O_2_^•−^: (A) quercetin; (B) Trolox; (C) CvExt. Figure S3: Absorption spectra of incubations containing BSA + CvExt after 30 days (A) or BSA + CvExt after 4 days (B). Figure S4: Absorption spectra of incubations containing BSA + glucose + CvExt after 30 days (A) or BSA + MG + CvExt after 4 days (B).

## Abbreviations

The following abbreviations are used in this manuscript:

AAPH	2,2′-azobis(2-amidinopropane) dihydrochloride
AG	Aminoguanidine
AGEs	Advanced glycation end products
BSA	Bovine serum albumin
ButFr	Butanolic fraction
CvExt	*Cissus verticillata* leaf hydroethanolic extract
DDA	Data-dependent acquisition
DM	Diabetes mellitus
DNPH	2,4-dinitrophenylhydrazine
EC_50_	50% effective concentration
ESI	Electrospray ionization
EtAcFr	Ethyl acetate fraction
Glu	Glucose
GNPS Molecular Networking	Global Natural Products Social Molecular Networking
HEtFr	Hydroethanolic fraction
HexFr	Hexane fraction
HISA	Herbarium of Ilha Solteira
LC-MS/MS	Liquid chromatography with tandem mass spectrometry
MG	Methylglyoxal
NADH	Nicotinamide adenine dinucleotide
NBT	Nitrotetrazolium blue
NuBBEDB	NuBBE Database
O_2_^•−^	Superoxide anion radical
PCO	Protein carbonyl groups
PLS-DA	Partial least-squares discriminant analysis
PMS	Phenazine metasulfate
QTOF	Quadrupole time-of-flight
ROO^•^	Peroxyl radical
ROS	Reactive oxygen species
SEM	Standard error of the mean
SisGen	National System for the Management of Genetic Heritage and Associated Traditional Knowledge
UPLC	Ultra-performance liquid chromatographer
