# Supplementary material for: Cissus verticillata Leaf Extract Decreases the Production of AGEs and ROS In Vitro
Source: Molecules. 2026 Feb 17;31(4):697. doi: 10.3390/molecules31040697 (PMC12943011; doi:10.3390/molecules31040697)
Supplement: Supplementary file 1 [file molecules-31-00697-s001.zip › molecules-4072483-supplementary.pdf]

Supplementary Table S1: Annotated metabolites from *Cissus verticilliata* in positive mode (ESI+).

MZmine ID	*m/z*	rt	Tentative metabolite	Evidence source	Evidence details	Class
29	223.989	0.62	Organosulfonic acid derivative	SIRIUS	Classyfire subclass: 98%	Organosulfonic acid derivative
52	156.0765	0.67	L-histidine	GNPS	MSMS: 110.0727	Amino acid
55	175.119	0.69	L-arginine	GNPS	MSMS: 158.092; 130.0985; 116.071	Amino acid
58	266.1593	0.69	Carbohydrate conjugate	SIRIUS	Classyfire subclass: 92%	Carbohydrate conjugate
68	130.05	0.73	Amino acid related	SIRIUS	NPC class: 98%	Amino acid
70	147.0763	0.72	Amino acid related	SIRIUS	NPC class: 98%	Amino acid
76	138.0552	0.77	Anthranilate	SIRIUS	TS: 82%	Anthranilate
80	175.1179	0.82	Amino acid related	SIRIUS	NPC class: 100%	Amino acid
84	136.0622	0.83	Adenin	SIRIUS	TS: 100%	Purine
93	121.0649	0.87	Phenylethanoid	SIRIUS	NPC class: 96%	Phenylethanoid
106	136.0617	1.01	Adenin	SIRIUS	TS:95%	Purine
110	136.062	1.06	Adenin	SIRIUS	TS: 100%	Purine
124	165.0544	1.23	p-Coumaric acid	Proposed	MSMS: 147.042; 119.047	Cinnamic acid
135	154.1341	1.31	Pyrazine/Piperazine alkaloid	SIRIUS	NPC class: 100%	Pyrazine/Piperazine alkaloid
140	152.0567	1.35	Primary amide	SIRIUS	NPC class: 82%	Primary amide
141	284.0985	1.35	Guanosine	GNPS	MSMS: 152.0565; 135.0310	Purine
159	154.134	1.77	Pyridine alkaloid	SIRIUS	NPC class: 87%	Pyridine alkaloid
174	103.0543	2.22	L-phenylalanine	Proposed	In-source fragment-175	Amino acid
175	120.0812	2.22	L-phenylalanine	Proposed	In-source fragment-176	Amino acid
176	166.0867	2.22	L-phenylalanine	GNPS	MSMS: 120.081	Amino acid

MZmine ID	*m/z*	rt	Tentative metabolite	Evidence source	Evidence details	Class
192	241.1695	2.49	Amine	SIRIUS	Cf subclass: 82%	Amine
213	188.0711	2.83	L-tryptophan	Proposed, FBMN-propagated	In-souce fragment-214	Amino acid
214	205.0973	2.83	L-tryptophan	GNPS	MSMS: 188.071; 146.060	Amino acid
218	146.0603	2.83	Indole-3-carboxaldehyde	GNPS	MSMS: 118.065	Tryptophan alkaloid
227	231.1701	2.96	Val-Leu	GNPS suspect library	MSMS: 185.174; 132.111	Amino acid
231	283.1745	2.99	Purine alkaloid	SIRIUS	NPC class: 87%	Purine
233	192.0767	3.02	Carbendazim	GNPS	MSMS: 160.051; 132.056	Pesticide (contaminant)
236	147.0439	3.05	p-coumaric acid [M-H2O+H]	GNPS	MSMS: 119.048	Cinnamic acid
239	355.1019	3.1	Neochlorogenic acid	GNPS suspect library	MSMS: 163.039	Cinnamic acid
241	163.0388	3.1	1H-pyrrolo[3,2-b]pyridine-5-carboxylic acid	GNPS	MSMS: 135.055; 117.044	Alkaloid
242	677.2077	3.12	3-p-coumaroylquinic acid related	Proposed, FBMN-propagated	MSMS: 467.131; 147.043	Cinnamic acid
256	217.0971	3.26	Tryptophan-related	Proposed, FBMN-propagated	Likely Trp oxidated to oxolactone	Amino acid
258	144.0804	3.26	Alkaloid	SIRIUS	NPC pathway: 100%	Alkaloid
262	595.1655	3.28	Vicenin 2	Proposed, FBMN-propagated	M+CH4O-282	O-glycosylated flavonoid
265	635.0665	3.31	Peptide alkaloid	SIRIUS	NPC superplass: 98%	Peptide alkaloid
268	927.1825	3.31	Amino acid related	SIRIUS	NPC pathway: 90%	Amino acid
279	179.0336	3.43	Esculetin	GNPS	MSMS: 151.041; 133.027	Coumarin
280	598.1559	3.43	Cyclamide	SIRIUS	NPC class: 91%	Amide
282	565.1549	3.52	Corymboside	GNPS	MSMS: 529.133;	O-glycosylated flavonoid

MZmine ID	*m/z*	rt	Tentative metabolite	Evidence source	Evidence details	Class
					511.132; 427.098	
287	339.1071	3.49	3-p-coumaroylquinic acid	GNPS	MSMS: 147.044	Cinnamic acid
289	147.044	3.5	p-coumaric acid	GNPS	MSMS: 119.049	Cinnamic acid
293	927.1815	3.55	Amino acid related	SIRIUS	NPC pathway: 97%	Amino acid
295	635.0668	3.55	a-aminoacid derivative	SIRIUS	Cf lvl6: 94%	Amino acid
303	516.3015	3.6	O-glycosyl compound	SIRIUS	Cf lvl6: 79%	O-glycosyl
305	565.155	3.61	Isoshaftoside	GNPS	MSMS: 547.154; 529.135; 511.120; 457.112	O-glycosylated flavonoid
312	279.1681	3.67	Bis-(2-ehtylhexyl) phthalate related	GNPS suspect library	MSMS: 201.047, 149.022	Phenolic acid (solvent contaminant)
314	681.2761	3.69	Oligopeptide	SIRIUS	NPC superclass: 100%	Oligopeptide
317	560.3269	3.73	Oligopeptide	SIRIUS	NPC superclass: 84%	Oligopeptide
318	279.1118	3.73	Triphenylphosphine oxide	GNPS	MSMS: 201.046	Phenylpropanoid (solvent contaminant)
323	147.0441	3.75	p-coumaric acid	GNPS	MSMS: 119.050	Cinnamic acid
333	279.1682	3.82	Bis-(2-ehtylhexyl) phthalate related	GNPS suspect library	MSMS: 201.044, 149.023	Phenolic acid (solvent contaminant)
334	453.3432	3.83	Phytosteroid (polar)	Proposed	Shared MSMS with bile acid	Phytosteroid (polar)
335	611.1611	3.85	Rutin	GNPS	MSMS: 303.050; 465.100	O-glycosylated flavonoid
338	279.1116	3.9	Triphenylphosphine oxide	GNPS	MSMS: 201.046	Phenylpropanoid (solvent contaminant)
344	465.1031	3.93	Myricetin-3-Xyloside	GNPS	MSMS: 319.045	O-glycosylated flavonoid
345	319.0452	3.93	Myricetin-3-Xyloside	Proposed	Myricetin (in-source fragment)	O-glycosylated flavonoid
366	595.1655	4.08	Cyanidin-3-O-rutinoside	GNPS	MSMS: 287.052;	O-glycosylated flavonoid

MZmine ID	*m/z*	rt	Tentative metabolite	Evidence source	Evidence details	Class
					259.055; 213.052	
369	736.4321	4.13	Oligopeptide	SIRIUS	NPC superclass: 100%	Oligopeptide
385	303.0497	4.29	Quercitrin	Proposed	Quecetrin (in-source fragment)	O-glycosylated flavonoid
386	449.1082	4.29	Quercitrin	GNPS	MSMS: 303.050	O-glycosylated flavonoid
393	224.1281	4.3	Anisole	SIRIUS	Classyfire subclass: 80%	Anisole
395	693.2764	4.32	Oligopeptide	SIRIUS	NPC superclass: 99%	Oligopeptide
408	606.369	4.42	Oligopeptide	SIRIUS	NPC superclass: 88%	Oligopeptide
416	633.3685	4.52	Oligopeptide	SIRIUS	NPC superclass: 100%	Oligopeptide
417	650.3953	4.52	Oligopeptide	SIRIUS	416+NH4	Oligopeptide
418	694.4205	4.6	Amino acid related	SIRIUS	Cf lvl5: 85%	Amino acid
420	179.1065	4.56	Benzenoid	SIRIUS	CSI:FingerID: 91.3%	Benzenoid
421	197.1171	4.56	Benzenoid	Proposed	420+H2O	Benzenoid
422	672.4083	4.56	Oligopeptide	SIRIUS	NPC superclass: 97%	Oligopeptide
429	433.1131	4.62	Kaempferol O-rhamnoside	GNPS	MSMS: 287:055	O-glycosylated flavonoid
430	287.0552	4.62	Kaempferol O-rhamnoside	GNPS	Kaempferol (in-source fragment)	O-glycosylated flavonoid
442	174.1848	4.69	Citronellol	Proposed, LOTUS db	MSMS: 156.173, M+NH4	Monoterpenoid
464	573.3077	5.15	Oligopeptide	SIRIUS	NPC superclass: 99%	Oligopeptide
476	692.3448	5.43	Oligopeptide	SIRIUS	NPC superclass: 100%	Oligopeptide
480	367.1324	5.52	Benzylpenicillin related	GNPS suspect library	delta m/z 32.026	β-lactam (contaminant)
488	367.1321	5.65	Benzylpenicillin related	GNPS suspect library	delta m/z 32.026	β-lactam (contaminant)
496	246.2426	5.74	Tetradecanoic acid	Proposed, LOTUS db	M+NH4	Fatty acid

MZmine ID	*m/z*	rt	Tentative metabolite	Evidence source	Evidence details	Class
516	401.2142	6.45	Polyol fatty acid	Proposed, SIRIUS	NPC: fatty acid, 89%; polyol, 69%	Polyol fatty acid
529	274.2744	6.67	Fatty acid conjugate	SIRIUS	NPC superclass: 90%	Fatty acid conjugate
538	318.3007	6.73	Sphingoid base	SIRIUS	NPC class: 95%	Sphingoid base
543	290.2687	6.76	Sphingoid base	GNPS suspect library	C16 sphingosine analog, delta m/z m/z 15.995	Sphingoid base
550	406.3524	6.79	Monoacylglycerol	SIRIUS	NPC class: 87%	Monoacylglycerol
557	272.2575	6.86	Unsaturated fatty acid	SIRIUS	NPC class: 83%	Unsaturated fatty acid
562	450.3779	6.86	Monoacylglycerol	SIRIUS	NPC class: 89%	Monoacylglycerol
570	288.2529	6.97	Fatty acid	GNPS, SIRIUS	MSMS: 277.200, NPC pathway: 99%	Fatty acid
584	316.2846	7.24	Fatty acid conjugate	SIRIUS	NPC superclass: 90%	Fatty acid conjugate
605	302.3053	7.53	Stearic acid	GNPS	MSMS: 284.295	Stearic acid
609	318.2999	7.54	Sphingoid base	SIRIUS	NPC class: 100%	Sphingoid base
611	346.3313	7.57	Fatty acid	SIRIUS	NPC pathway: 100%	Fatty acid
623	300.2892	7.71	Unsaturated fatty acid	SIRIUS	NPC class: 91%	Unsaturated fatty acid
626	316.2844	7.8	Fatty acid	GNPS, SIRIUS	MSMS: 255.231, NPC pathway: 97%	Fatty acid
630	437.1934	7.82	Amine	SIRIUS	Cf subclass: 85%	Amine
639	351.2523	7.87	Monoacylglicerol	SIRIUS	NPC class: 100%	Monoacylglicerol
642	288.2529	7.89	Fatty acid	GNPS, SIRIUS	MSMS: 227.203, 106.084, NPC pathway: 97%	Fatty acid
680	330.3367	8.32	Sphingoid base	GNPS suspect library, SIRIUS	NPC class: 100%	Sphingoid base
683	374.3626	8.35	Monoacylglicerol	SIRIUS	NPC class: 80%	Monoacylglicerol

MZmine ID	*m/z*	rt	Tentative metabolite	Evidence source	Evidence details	Class
691	437.1404	8.37	Purine nucleoside	SIRIUS	NPC class: 87%	Purine
696	277.2157	8.41	9-HOTrE	GNPS	MSMS: 149.132, 135.116, 121.101	Fatty acid
699	553.4254	8.42	(2R)-beta,beta-Caroten-2-ol	GNPS	MSMS: 535.409, 517.410	Carotenoid
710	344.3158	8.55	Fatty acid	GNPS, SIRIUS	MSMS: 283.261, NPC pathway: 97%	Fatty acid
723	504.3836	8.66	Oligopeptide	SIRIUS	NPC superclass: 81%	Oligopeptide
730	315.1925	8.78	12-hydroxyjasmonic acid related	FBMN-propagated	[(735)+Na]	Fatty acid
734	454.293	8.78	1-Oleoyl-sn-glycero-3-phosphoethanolamine related	GNPS suspect library	MSMS: 436.284; 313.271	Glycerophospholipid
735	293.2106	8.78	12-hydroxyjasmonic acid related	GNPS suspect library	MSMS: 275.199; 149.095	Fatty acid
742	327.0781	8.85	Triphenyl phosphate	GNPS	MSMS: 251.046	Phenylpropanoid (solvent contaminant)
748	496.3398	8.91	PC(0:0/16:0)	GNPS	MSMS: 478.327, 184.074, 104.106	Fatty acid
752	224.1074	8.95	Mefenamic acid	GNPS	MSMS: 209.083, 181.088, 152.062	Phenolic acid (solvent contaminant)
758	410.0875	8.98	Pyraclostrobin	FBMN-propagated	760+Na	Pesticide (contaminant)
760	388.1059	8.98	Pyraclostrobin	GNPS	MSMS: 296.058, 194.081, 163.062	Pesticide (contaminant)
764	358.3679	9.04	Sphingoid base	SIRIUS	NPC class: 98%	Sphingoid base
767	316.284	9.06	Fatty acid	GNPS, SIRIUS	NPC pathway: 99%	Fatty acid
771	501.303	9.09	a-aminoacid derivative	SIRIUS	Cf lvl6: 82%	Amino acid
786	451.1558	9.12	Dibutoxyethoxyethyl adipate related	FBMN-propagated	977 analog, delta m/z 15.861,	Fatty ester

MZmine ID	*m/z*	rt	Tentative metabolite	Evidence source	Evidence details	Class
					indicating an extra O	
807	246.111	9.18	Adipate related	Proposed	825-same RT	Fatty ester
808	505.3486	9.18	Adipate related	FBMN-propagated	803-delta m/z 188.152	Fatty ester
823	317.1961	9.18	Dibutoxyethoxyethyl adipate	Proposed	825-In-source fragment	Fatty ester
825	435.2949	9.18	Dibutoxyethoxyethyl adipate	GNPS	MSMS: 317.196	Fatty ester
826	452.322	9.18	Dibutoxyethoxyethyl adipate	GNPS	MSMS: 317.197	Fatty ester
871	306.2787	9.19	Kaurenol	Proposed, LOTUS db	M+NH4	Deterpenoid
877	372.347	9.24	Fatty acid	GNPS, SIRIUS	MSMS: 311.293, NPC pathway: 100%	Fatty acid
959	673.3763	9.5	Glycosilated long-chain polyketide	Proposed, SIRIUS	NPC class: polyketide; 75%, carbohydrate conjugate; 82%	Polyketide
965	629.3509	9.57	Oligopeptide	SIRIUS	NPC superclass: 97%	Oligopeptide
977	279.1591	9.62	Dibutylphthalate	GNPS	MSMS: 149.024	Phenolic acid (solvent contaminant)
979	212.1436	9.63	L-phenylalanine related	FBMN-propagated	MSMS: 120.081	Amino acid
1049	349.2355	9.88	Phtalate contaminant	Proposed, FBMN-propagated	delta m/z 119.073	Phenolic acid (solvent contaminant)
1062	643.367	9.91	Macrotetrolide	SIRIUS	NPC class: 92%	Macrotetrolide (bacterial)
1081	324.2892	9.96	Endocannabinoid	Proposed, CSI:FingerID fingerprinting, FBMN-propagated	delta m/z 2.015, extra insaturation	Endocannabinoid
1167	565.0971	10.19	Cyclamide	SIRIUS	NPC class: 84%	Amide
1207	623.2506	10.33	Pheophorbide A related	Proposed, FBMN-propagated	delta m/z 13.98	Chlorophyll-like
1233	484.4723	10.38	Tetracosanoic-acid_1,4-cyclohexanediol	GNPS	MSMS: 283.262, 255.232, 288.231	Fatty acid

MZmine ID	*m/z*	rt	Tentative metabolite	Evidence source	Evidence details	Class
1254	300.2896	10.4	Oleic acid [M+NH4]	Proposed	MSMS: 283.264	Unsaturated fatty acid
1272	341.2661	10.42	Fatty alcohol	SIRIUS	NPC class: 88%	Fatty alcohol
1322	607.2553	10.59	Pheophorbide A related	Proposed, FBMN-propagated	delta m/z 13.99, consistent with C-chain elongation	Chlorophyll-like
1323	439.3568	10.59	Oleanolic acid	GNPS	MSMS: 191.181; 203.179	Triterpene
1328	468.4771	10.6	Fatty amide	SIRIUS	NPC superclass: 94%	Fatty amide
1329	251.0472	10.6	Diphenylphosphate	GNPS suspect library, proposed	MSMS: 152.061	Phenylpropanoid (solvent contaminant)
1346	383.2423	10.61	Amino acid related	SIRIUS	NPC Pathway: 85%	Amino acid
1352	379.2159	10.61	Amine	SIRIUS	Cf subclass: 99%	Amine
1372	326.3046	10.67	Endocannabinoid	SIRIUS	NPC class 90%	Endocannabinoid
1395	512.5037	10.74	Fatty amide	GNPS, SIRIUS	NPC superclass: 98%	Fatty amide
1401	398.2324	10.75	Diethylamino hydroxybenzoyl hexyl benzoate	GNPS	MSMS: 149.024	Phenylpropanoid (solvent contaminant)
1440	256.2636	10.83	Terpenoid	SIRIUS	NPC Pathway: 97%	Terpenoid
1474	259.2045	10.87	Terpenoid	Proposed, FBMN-propagated	delta m/z 2.94	Terpenoid
1483	607.2554	10.89	Pheophorbide A related	Proposed, FBMN-propagated	delta m/z 13.99, consistent with C-chain elongation	Chlorophyll-like
1599	637.306	11.03	Secoiridoid monoterpenoid	SIRIUS	NPC class: 98%	Terpenoid
1600	659.2877	11.03	Oligopeptide	SIRIUS	NPC superclass: 94%	Oligopeptide
1626	256.2633	11.06	Fatty alcohol	SIRIUS	NPC class: 81%	Fatty alcohol

MZmine ID	*m/z*	rt	Tentative metabolite	Evidence source	Evidence details	Class
1650	524.3825	11.09	Small peptide	SIRIUS	NPC superclass: 100% (contains Leu)	Amino acid
1666	625.266	11.09	Pheophorbide A related	Proposed, FBMN-propagated	delta m/z 28.03	Chlorophyll-like
1697	609.2711	11.05	10S-Hydroxypheophorbide A	GNPS	MSMS: 591.261; 531.2449; 559.2343	Chlorophyll-like
1703	465.357	11.13	Diradylglycerol	SIRIUS	Glycerolipid 87%; El Gordo: DG(14:0_9:0)	Glycerolipid
1744	540.5348	11.17	4-acetylbutyric-acid_1-octacosanol	GNPS	MSMS: 311.294, 284.294, 256.263	Fatty acid
1785	609.271	11.25	10S-Hydroxypheophorbide A	GNPS	MSMS: 591.261; 531.2449; 559.2343	Chlorophyll-like
1814	282.2783	11.27	Fatty amide	SIRIUS	NPC superclass: 100%	Fatty amide
1860	457.3652	11.32	Triterpenoid	Proposed	MSMS: 291.162	Triterpenoid
1867	667.2757	11.33	Pheophorbide A related	Proposed, FBMN-propagated	delta m/z 16.00	Chlorophyll-like
1884	282.221	11.35	Carotenoid	SIRIUS, FBMN-propagated	NPC superclass: 75%	Carotenoid
1915	703.4759	11.39	Diacylglycerol	SIRIUS	NPC superclass: 94%	Diacylglycerol
1919	185.115	11.4	Fatty alcohol	SIRIUS	NPC class: 82%	Fatty alcohol
1951	328.321	11.43	Endocannabinoid	Proposed, CSI:FingerID fingerprinting, FBMN-propagated		Endocannabinoid
1956	593.2762	11.44	Pheophorbide A	GNPS	MSMS: 533.254	Chlorophyll-like
1983	673.2628	11.47	Pheophorbide A related	Proposed, FBMN-propagated	delta m/z 13.98	Chlorophyll-like

MZmine ID	*m/z*	rt	Tentative metabolite	Evidence source	Evidence details	Class
1984	651.2809	11.47	Pheophorbide A related	Proposed, FBMN-propagated	delta m/z 42.01	Chlorophyll-like
2051	354.2986	11.6	1-Monolinoleoyl-rac-glycerol related	GNPS suspect library	delta m/z -0.984	Glycerolipid
2059	431.3365	11.65	Wax diester	SIRIUS	NPC subclass: 88%	Fatty ester
2060	448.3634	11.65	Wax diester	SIRIUS	NPC class: 80%	Fatty ester
2078	593.2757	11.72	Pheophorbide A	GNPS	MSMS: 533.254	Chlorophyll-like
2088	326.3049	11.8	Endocannabinoid	Proposed, CSI:FingerID fingerprinting, FBMN-propagated	Isomer-1372	Endocannabinoid
2091	635.2852	11.81	Pheophorbide A related	Proposed, FBMN-propagated, suspect-library	delta m/z 42.009, consistent with chain elongation	Chlorophyll-like
2098	284.2938	12.03	Fatty acid	SIRIUS	NPC pathway: 98%	Fatty acid
2102	653.2968	12.1	Pheophorbide A related	Proposed, FBMN-propagated	delta m/z 13.98	Chlorophyll-like
2108	637.3014	12.22	Pheophorbide A related	Proposed, FBMN-propagated	delta m/z 28.0303, consistent with +C2H4	Chlorophyll-like
2110	659.2836	12.23	Pheophorbide A related	Proposed, FBMN-propagated	[M+Na]	Chlorophyll-like
2118	442.3885	12.42	Fatty acid conjugate	SIRIUS	Cf subclass: 83%	Fatty acid conjugate
2121	391.2842	12.5	Bis(2-ethylhexyl) phthalate	GNPS	MSMS: 149.023	Phenolic acid (solvent contaminant)
2128	621.3067	12.54	Pheophorbide A related	GNPS, FBMN-propagated, suspect-library	delta m/z 28.04, consistent with +C2H4	Chlorophyll-like
2131	579.3869	12.54	Oligopeptide	SIRIUS	NPC superclass: 87%	Oligopeptide
2132	765.4761	12.61	Lipopeptide	SIRIUS	NPC class: 85%	Lipopeptide

MZmine ID	*m/z*	rt	Tentative metabolite	Evidence source	Evidence details	Class
2136	663.4542	12.83	Oligopeptide	SIRIUS	NPC superclass: 91%	Oligopeptide
2148	702.214	13.08	Solvent contaminat	GNPS	Known contaminant	Contaminant
2152	394.3464	13.8	Carotenoid	FBMN-propagated, SIRIUS	NPC superclass: 99%	Carotenoid

MZmine ID was automatically generated during LC-MS/MS processing. Each spectrum can be accessed searching the ID in the GNPS2 job (https://gnps2.org/status?task=c9a532e273c94bfd85c234e7af76a8e2; "Cluster Summary"); *m/z*: detected precursor ion; rt: retention time. SIRIUS calculations files are available in the supplementary materials.

Supplementary Table S2: Annotated metabolites from *Cissus verticilliata* in negative mode (ESI$^-$).

MZmine ID	*m/z*	rt	Tentative metabolite	Evidence source	Evidence details	Class
9	173.1026	0.68	Homoarginin	SIRIUS	Tanimoto: 78%	Amino acid
12	131.0447	0.72	Homoaspartic acid	SIRIUS	Tanimoto: 93.4%	Amino acid
39	191.018	0.95	Citric acid	GNPS	MSMS: 111.006, 102.947	Hydroxy fatty acid
45	191.0181	1.12	Citric acid	GNPS	MSMS: 111.007	Hydroxy fatty acid
47	289.0662	1.14	Uracil arabinoside	GNPS	[(111)+HCOOH-H], MSMS: 243.063, 200.055	Nucleoside
54	191.0176	1.53	Citric acid	GNPS	MSMS: 146.896, 111.008, 102.946	Hydroxy fatty acid
59	355.1015	2.22	Isosulochrin	GNPS	MSMS: 181.0486, 121.0284	Aromatic polyketides
63	315.071	2.44	Benzoic acid + 2O_ O-Hex	GNPS	MSMS: 152.009, 108.021	Phenolic acid
64	218.1018	2.49	Pantothenic acid-B5	GNPS	MSMS: 218.101, 146.083	Dipeptide
66	355.1012	2.54	Chlorogenic acid	GNPS	MSMS: 191.052	Cinnamic acid
69	315.0713	2.65	Benzoic acid + 2O_ O-Hex	GNPS	MSMS: 153.018, 109.026	Phenolic acid
72	153.0198	2.74	Gentisate	GNPS	MSMS: 109.027	Phenolic acid
74	353.0861	2.81	Neochlorogenic acid	GNPS	MSMS: 191.054, 179.033, 135.043	Cinnamic acid
75	371.0971	2.86	4-O-beta-D-glucosyl-4-coumaric acid	GNPS	[M+HCOOH-H], MSMS: 163.038, 119.049	Coumarin
78	355.0654	2.9	Coumaroyl + C6H9O8	GNPS	MSMS: 209.026, 191.018	Cinnamic acid
79	203.0811	2.93	Tryptophan	GNPS	MSMS: 116.048	Amino acid
90	355.0655	3.13	Coumaroyl + C6H9O8	GNPS	MSMS: 209.027, 191.018	Cinnamic acid
94	337.0915	3.16	Isochlorogenic acid B related	GNPS	MSMS: 191.056, 163.039, 119.052, delta m/z -15.995	Cinnamic acid
97	191.0546	3.2	Kinic acid	GNPS	Tanimoto: 96.9%	Phenolic acid
98	353.0864	3.2	Chlorogenic acid	GNPS	MSMS: 191.055	Cinnamic acid
103	163.0387	3.23	Coumaric acid	GNPS	MSMS: 119.049	Cinnamic acid
104	325.0915	3.24	1-O-(4-Coumaroyl)-beta-D-glucose	GNPS	MSMS: 163.012, 119.047	Cinnamic acid
114	153.0179	3.34	Gentisate	GNPS	MSMS: 108.020	Phenolic acid

MZmine ID	*m/z*	rt	Tentative metabolite	Evidence source	Evidence details	Class
116	355.0657	3.35	Coumaroyl + C6H9O8	GNPS	MSMS: 209.028, 191.016, 163.099	Cinnamic acid
121	593.1501	3.39	Apigenin_Hex_Hex	GNPS, Proposed	MSMS: 473.107, 383.071,353.065	O-glycosylated flavonoid
133	353.0864	3.48	Chlorogenic acid	GNPS	MSMS: 191.054	Cinnamic acid
139	177.0178	3.52	6,7-Dihydroxycoumarin	GNPS	MSMS: 133.027, 105.032	Coumarin
145	337.0918	3.6	Chlorogenic acid related	GNPS	MSMS: 191.054, 173.044, 119.049, delta m/z -15.995	Cinnamic acid
146	191.0546	3.6	Kinic acid	GNPS	Tanimoto: 95%	Phenolic acid
148	563.1392	3.62	Apigenin_Pen_Hex	GNPS, Proposed	MSMS: 473.097, 443.095, 383.075	O-glycosylated flavonoid
152	625.1399	3.66	Myricetin 3-O-rutinoside	GNPS	MSMS: 316.019, 271.024	O-glycosylated flavonoid
158	563.1395	3.69	Apigenin_Pen_Hex	GNPS, Proposed	MSMS: 473.107, 443.096, 383.074	O-glycosylated flavonoid
159	355.1018	3.7	Chlorogenic acid	GNPS	MSMS: 163.040, 145.028	Cinnamic acid
163	367.1012	3.75	3-O-Feruloylquinic acid	GNPS	MSMS: 191.056, 173.042, 134.038	Cinnamic acid
164	479.0817	3.75	Myricetin-3-O-galactoside	GNPS	MSMS: 316.020, 271.023	O-glycosylated flavonoid
176	191.0546	3.85	Kinic acid	SRIUS	Tanimoto: 95%	Phenolic acid
177	337.0916	3.85	Chlorogenic acid related	GNPS	MSMS: 191.054, delta m/z -15.995	Cinnamic acid
179	431.1908	3.87	Corchoionoside C	GNPS	MSMS: 385.183, 205.120, 119.033	Apocarotenoid
182	609.1453	3.92	Rutin	GNPS	MSMS: 300.025	O-glycosylated flavonoid
184	327.0854	3.96	Coumaric acid	GNPS	MSMS: 119.048	Cinnamic acid
189	927.1844	4.03	Myricitrin	GNPS	MSMS: 463.087, 316.021, 2M-H	O-glycosylated flavonoid
191	463.0879	4.03	Myricitrin	GNPS	MSMS: 316.021, 271.023	O-glycosylated flavonoid
192	271.0229	4.03	Myricitrin	Proposed	Myricitrin in-source fragment	O-glycosylated flavonoid
197	163.0383	4.08	3-Hydroxycinnamic acid	GNPS	MSMS: 119.049	Cinnamic acid
205	593.1499	4.18	Cyanidin-3-O-rutinoside	GNPS	MSMS: 285.038	O-glycosylated flavonoid
212	163.0386	4.25	Coumaric acid	GNPS	MSMS: 119.049	Cinnamic acid
219	303.0492	4.33	Taxifolin	SIRIUS	Tanimoto: 83%	Flavonoid

MZmine ID	*m/z*	rt	Tentative metabolite	Evidence source	Evidence details	Class
225	271.0233	4.4	Quercitrin	Proposed	Quercitrin in-souce fragment	O-glycosylated flavonoid
227	447.0926	4.4	Quercitrin	GNPS	MSMS: 300.026, 271.023	O-glycosylated flavonoid
228	895.1945	4.4	Quercitrin	GNPS	MSMS: 300.027, 2M-H	O-glycosylated flavonoid
231	477.1023	4.43	2-(3,4-dihydroxyphenyl)-5,8-dihydroxy-7-methoxy-3-[(2S,3R,4R,5R,6S)-3,4,5-trihydroxy-6-methyloxan-2-yl]oxychromen-4-one	GNPS	MSMS: 330.036, 315.013, 287.167, 271.059	O-glycosylated flavonoid
252	243.122	4.67	4-oxododecanedioic acid	GNPS	MSMS: 255.113, 207.103, 199.135, 181.119	Fatty acid
254	187.0962	4.7	Azelaic acid	GNPS	MSMS: 125.097	Fatty acid
255	863.204	4.72	Afzelin	Proposed	Afzelin 2M-H	O-glycosylated flavonoid
256	285.0384	4.72	Afzelin	Proposed	Kaempferol unity as in-source fragment	O-glycosylated flavonoid
257	477.1027	4.72	Afzelin	Proposed	[M+HCOOH-H]	O-glycosylated flavonoid
258	431.0974	4.72	Afzelin	GNPS	MSMS: 285.038, 255.028, 227.035	O-glycosylated flavonoid
262	499.0846	4.73	Afzelin related	GNPS suspect library	MSMS: 285.040, kaempferol backbone	O-glycosylated flavonoid
271	287.0545	4.83	Dihydrokaempferol	GNPS	MSMS: 259.058, 177.055, 151.004, 125.023	Flavonoid
277	471.1281	4.89	2-({6-O-[(2E)-3-(4-Hydroxyphenyl)-2-propenoyl]-beta-D-glucopyranosyl}oxy)-3-phenylacrylic acid	GNPS	MSMS: 163.039, 145.030, 119.052	Cinnamic acid
278	463.0864	4.89	Myricitrin	GNPS	MSMS: 316.021, 271.023	O-glycosylated flavonoid
284	463.0866	4.95	Myricitrin	GNPS	MSMS: 316.026, 271.024	O-glycosylated flavonoid
343	711.395	5.52	Niga-ichigoside F1	Proposed	MSMS: 503.342	Saponin
352	695.3994	5.69	Kaji-ichigoside F1	Proposed	MSMS: 487.340, 469.341	Saponin
356	655.4412	5.82	(10E,15E)-9,12,13-trihydroxyoctadeca-10,15-dienoic acid	GNPS	MSMS: 327.217	Fatty acid

MZmine ID	*m/z*	rt	Tentative metabolite	Evidence source	Evidence details	Class
360	327.2167	5.83	FA 18:2+3O	GNPS	MSMS: 229.143, 211.132, 171.101	Fatty acid
361	215.1267	5.86	Undecanedioic acid	SIRIUS	Tanimoto: 79.5%	Fatty acid
375	227.127	6.14	Traumatic acid	GNPS	MSMS: 183.138	Triterpene
376	329.2318	6.14	9,12,13,TriHODE related	GNPS suspect library	MSMS: 229.142, 211.132, 171.101, delta m/z 2.016	Fatty acid
389	327.2163	6.52	FA 18:1+3O	GNPS	MSMS: 171.102	Fatty acid
397	327.2159	6.83	9S-HpOTrE related	GNPS	MSMS: 171.101, 155.110, delta m/z 18.01	Fatty acid
406	311.1833	7.13	5-trans-Prostaglandin D2 related	GNPS	MSMS: 293.172, 275.166, 267.193, delta m/z -4.011	Fatty acids
431	721.3637	7.78	Glc-Glc-octadecatrienoyl-sn-glycerol	GNPS	MSMS: 397.134, 277.216	Glycerolipid
438	283.1357	7.91	Steroid-like	GNPS suspect library	MSMS: 183.010, delta m/z -56.062	Terpenoid
441	721.3644	7.95	Glc-Glc-octadecatrienoyl-sn-glycerol	GNPS	MSMS: 397.132, 277.215	Glycerolipid
444	593.2707	7.99	PI(18:1/0:0)	GNPS	MSMS: 315.059, 277.211, 241.010, 152.994, delta m/z -4.024	Glycerophospholipids
450	297.1514	8.14	7-(geranyloxy)coumarin	GNPS	MSMS: 197.026, 183.011	Coumarin
459	297.1517	8.27	7-(geranyloxy)coumarin	GNPS	MSMS: 197.026, 183.012	Coumarin
468	297.152	8.43	Decylbenzenesulfonic acid	GNPS	MSMS: 183.013	Aromatic polyketide
475	309.2052	8.57	methyl (2E,4E,8E)-7,13-dihydroxy-4,8,12-trimethyltetradeca-2,4,8-trienoate	GNPS	MSMS: 197.023, 183.009	Linear polyketides
476	297.1516	8.6	Decylbenzenesulfonic acid	GNPS	MSMS: 183.011	Aromatic polyketide
482	434.9299	8.69	Ethiprole related	GNPS suspect library	MSMS: 329.958, 277.953, 249.956, delta m/z 39.958	Pesticide (contaminant)
483	309.2058	8.69	methyl (2E,4E,8E)-7,13-dihydroxy-4,8,12-	GNPS	MSMS: 197.027, 183.011	Linear polyketides

MZmine ID	*m/z*	rt	Tentative metabolite	Evidence source	Evidence details	Class
			trimethyltetradeca-2,4,8-trienoate			
486	559.3109	8.73	MGMG 18:3	GNPS	MSMS: 277.222, 253.091	Glycerolipids
497	540.3297	9.06	LPC 16:0	GNPS	MSMS: 480.304, 255.231	Glycerophospholipids
499	450.925	9.12	Fipronil sulfone	GNPS	MSMS: 414.947, 281.991, 243.989	Pesticide
524	271.2262	10.64	16-Hydroxyhexadecanoic acid	SIRIUS	Tanimoto: 73.3%	Fatty acid
529	501.3575	10.72	Usolic/oleanolic acid	Proposed	[M+HCOOH-H]	Triterpene
532	455.3513	10.91	Usolic/oleanolic acid	Proposed	[M-H], taxonomical plausability, low-quality MSMS	Triterpene
561	471.3469	12.23	Echinocystic acid	GNPS	MSMS: 427.356, 409.346, 393.314	Triterpene

MZmine ID was automatically generated during LC-MS/MS processing. Each spectrum can be accessed searching the ID in the GNPS2 job (https://gnps2.org/status?task=a8e38539ed1a437b9624c8045bb7c706; “Cluster Summary”); *m/z*: detected precursor ion; rt: retention time. SIRIUS calculations files are available in the supplementary materials.

Supplementary Table S3: Complete Spearman correlation analysis of anti-AGE activity in the most active fractions of *Cissus verticillata* leaf hydroethanolic extract (CvExt) in positive mode (ESI^+).

Compound	HexFr	EtAcFr	ButFr
(1110)	0	0,48245	0,9649
1-Oleoyl-sn-glycero-3-phosphoethanolamine related (734)	0,17321	0,48245	0,9649
Benzenoid (420)	0,9649	0,9649	0,9649
Cyanidin-3-O-rutinoside (366)	0	0,9113	0,9649
Esculetin (279)	0	0,9649	0,9649
Fatty amide (1328)	0,33029	0,53606	0,9649
O-glycosyl compound (303)	0	0	0,9649
PC(0:0/16:0) (748)	0	0,53606	0,9649
Tetracosanoic-acid_1,4-cyclohexanediol (1233)	0	0,48245	0,9649
Triphenylphosphine oxide (338)	0	0,48245	0,9649
(336)	0,16082	0,47633	0,95266
(739)	-0,17321	-0,05505	0,95266
(244)	-0,48245	-0,47434	0,94868
(261)	-0,48245	0,57975	0,94868
(272)	-0,48245	0,47434	0,94868
(286)	-0,48245	0,47434	0,94868
(324)	-0,48245	0,47434	0,94868
(440)	-0,47434	-0,47434	0,94868
(558)	-0,48245	-0,47434	0,94868
(610)	-0,47434	-0,47434	0,94868
1H-pyrrolo[3,2-b]pyridine-5-carboxylic acid (241)	-0,48245	-0,15811	0,94868
3-p-coumaroylquinic acid (287)	-0,48245	0,52705	0,94868
Adenin (110)	-0,48245	-0,47434	0,94868
Adenin (84)	-0,48245	0,94868	0,94868
Alkaloid (258)	-0,48245	0,47434	0,94868
Benzenoid (421)	0,94868	0,94868	0,94868
Bis-(2-ehtylhexyl) phthalate related (312)	-0,48245	0,47434	0,94868
Bis-(2-ehtylhexyl) phthalate related (333)	-0,48245	0,15811	0,94868

**Compound**	**HexFr**	**EtAcFr**	**ButFr**
Corymboside (282)	-0,48245	-0,05271	0,94868
Dibutoxyethoxyethyl adipate (826)	0,94868	0,94868	0,94868
Endocannabinoid (1951)	0,94868	0,47434	0,94868
Fatty acid (570)	-0,47633	0,52705	0,94868
Fatty acid (626)	-0,48245	0,47434	0,94868
Fatty acid (710)	0,47434	0,52705	0,94868
Fatty acid (877)	0,47434	0,57975	0,94868
Fatty amide (1395)	0,47434	0,47434	0,94868
Isoshaftoside (305)	-0,48245	-0,26352	0,94868
Myricetin-3-Xyloside (344)	-0,48245	0,94868	0,94868
Myricetin-3-Xyloside (345)	-0,48245	0,94868	0,94868
Oleic acid [M+NH4] (1254)	0,94868	0,47434	0,94868
Oligopeptide (395)	-0,48245	0,47434	0,94868
p-coumaric acid (289)	-0,48245	0,47434	0,94868
p-coumaric acid (323)	-0,48245	0,57975	0,94868
Quercitrin (385)	-0,47434	0,94868	0,94868
Quercitrin (386)	-0,48245	0,94868	0,94868
Rutin (335)	-0,33029	0,94868	0,94868
Tetradecanoic acid (496)	-0,47434	-0,47434	0,94868
Tryptophan-related (256)	-0,48245	0,47434	0,94868
Unsaturated fatty acid (557)	-0,47434	-0,47434	0,94868
Unsaturated fatty acid (623)	-0,48245	-0,47434	0,94868
Val-Leu (227)	-0,48245	-0,47434	0,94868
Vicenin 2 (262)	-0,48245	-0,47434	0,94868
(2087)	0,9083	0	0,9083
(246)	-0,53606	0,57975	0,89598
Purine alkaloid (231)	-0,47434	-0,47434	0,89598
(1735)	0,43301	0	0,8454
(225)	0	0,8454	0,8454
(452)	0,8454	0,8454	0,8454
(453)	0,64952	0,8454	0,8454
(511)	0,8454	0,8454	0,8454
(512)	0,8454	0,8454	0,8454

Compound	HexFr	EtAcFr	ButFr
Adipate related (807)	0,8454	0,8454	0,8454
Adipate related (808)	0,8454	0,8454	0,8454
Dibutoxyethoxyethyl adipate (823)	0,8454	0,8454	0,8454
Glycosilated long-chain polyketide (959)	0,8454	0,8454	0,8454
Polyol fatty acid (516)	0,8454	0,8454	0,8454
Terpenoid (1440)	0,8454	0	0,8454
(1179)	-0,36893	0,26352	0,84327
(498)	-0,57975	-0,57975	0,84327
4-acetylbutyric-acid_1-octacosanol (1744)	0,47434	0,47434	0,84327
Neochlorogenic acid (239)	-0,48245	0,15811	0,84327
Purine nucleoside (691)	-0,47434	0,47434	0,84327
(775)	0,9649	0,9649	0,80408
a-aminoacid derivative (295)	0	0,48245	0,80408
(364)	-0,05505	0,15878	0,79388
Amino acid related (293)	-0,17321	0,47633	0,79388
Oligopeptide (317)	0,055048	0,055048	0,79388
(143)	-0,64327	-0,63246	0,79057
(2100)	-0,48245	-0,47434	0,79057
Kaempferol O-rhamnoside (429)	-0,48245	0,94868	0,79057
Oligopeptide (314)	-0,64327	0,42164	0,79057
(707)	-0,17321	0,47633	0,74096
(188)	-0,48245	0,15811	0,73786
(687)	-0,47434	-0,47434	0,73786
Kaempferol O-rhamnoside (430)	-0,48245	0,94868	0,73786
L-phenylalanine (174)	-0,48245	-0,47434	0,73786
L-phenylalanine (176)	-0,48245	-0,47434	0,73786
(853)	0,69282	0,69282	0,69282
(356)	0,053606	0,37048	0,68803
(85)	-0,48245	-0,47434	0,68516
Indole-3-carboxaldehyde (218)	-0,48245	-0,47434	0,68516
L-phenylalanine (175)	-0,48245	-0,47434	0,68516
L-tryptophan (213)	-0,48245	-0,47434	0,68516
L-tryptophan (214)	-0,48245	-0,47434	0,68516

Compound	HexFr	EtAcFr	ButFr
(2005)	0,48245	0,48245	0,64327
Dibutoxyethoxyethyl adipate (825)	0,64327	0,64327	0,64327
Carotenoid (1884)	-0,10541	-0,52705	0,63246
Cyclamide (1167)	-0,47633	0,26352	0,63246
Dibutoxyethoxyethyl adipate related (786)	-0,47434	0,26352	0,63246
(668)	0,86603	0,5636	0,62622
(2007)	0,48245	0,48245	0,58966
Fatty acid conjugate (584)	0,48245	0,9649	0,58966
(402)	0	0,33029	0,53606
(1139)	0,48245	0,48245	0,48245
(1887)	0,17321	0,48245	0,48245
(259)	0	0,48245	0,48245
(412)	0	0,33029	0,48245
(441)	0	0	0,48245
(465)	0,48245	0,9649	0,48245
(490)	0,33029	0,9649	0,48245
(493)	0,48245	0,9649	0,48245
(544)	0,9649	0,9649	0,48245
(586)	0,69687	0,48245	0,48245
10S-Hydroxypheophorbide A (1785)	0,9649	0,48245	0,48245
Amino acid related (418)	0	0	0,48245
Endocannabinoid (1081)	0,9649	0,48245	0,48245
Fatty acid (2098)	0,48245	0	0,48245
Oligopeptide (408)	0,17321	0,48245	0,48245
Oligopeptide (416)	0	0	0,48245
Oligopeptide (417)	0	0,48245	0,48245
Oligopeptide (422)	0	0,17321	0,48245
Pheophorbide A (2078)	0,48245	0,48245	0,48245
Pheophorbide A related (1207)	0,48245	0,48245	0,48245
Pheophorbide A related (1322)	0,48245	0,48245	0,48245
Pheophorbide A related (1483)	0,33029	0,48245	0,48245
Pheophorbide A related (1666)	0,9649	0,58966	0,48245
Pheophorbide A related (1867)	0,9649	0,64327	0,48245

**Compound**	**HexFr**	**EtAcFr**	**ButFr**
Pheophorbide A related (1983)	0,9649	0,48245	0,48245
Pheophorbide A related (1984)	0,9649	0,48245	0,48245
Pheophorbide A related (2091)	0,48245	0,48245	0,48245
Solvent contaminat (2148)	0,9649	0,58966	0,48245
Triterpenoid (1860)	0,9649	0,64327	0,48245
(117)	-0,86603	-0,86603	0,47633
10S-Hydroxypheophorbide A (1697)	0,95266	0,47633	0,47633
9-HOTrE (696)	0,95266	0,47633	0,47633
(1262)	0,26352	-0,47434	0,47434
(2037)	0,47434	-0,15811	0,47434
(2107)	0,47434	0,47434	0,47434
(313)	-0,48245	0,15811	0,47434
(444)	-0,94868	-0,94868	0,47434
(497)	-0,94868	-0,94868	0,47434
(532)	-0,94868	-0,94868	0,47434
(678)	-0,47434	-0,47434	0,47434
(766)	-0,36893	-0,36893	0,47434
Adenin (106)	-0,9649	0,47434	0,47434
Amino acid related (268)	-0,48245	0,47434	0,47434
Peptide alkaloid (265)	-0,48245	0,47434	0,47434
Pheophorbide A (1956)	0,47434	0,47434	0,47434
Pyridine alkaloid (159)	-0,48245	0,47434	0,47434
Sphingoid base (609)	0,47434	0,47434	0,47434
Sphingoid base (764)	0,47434	-0,15811	0,47434
a-aminoacid derivative (771)	0,8454	0,8454	0,43301
Endocannabinoid (2088)	0,8454	0	0,43301
Macrotetrolide (1062)	0,8454	0,8454	0,43301
Citronellol (442)	-0,94868	-0,94868	0,36893
Fatty acid (642)	-0,47434	-0,47434	0,36893
(1578)	0	0	0,36084
(1111)	0,9649	0,64327	0,33029
(1273)	0,47434	-0,47434	0,26352
(396)	0	0	0,17321

Compound	HexFr	EtAcFr	ButFr
(705)	0,9649	0,80408	0,17321
(737)	0,9649	0,48245	0,17321
Monoacylglicerol (639)	0,9649	0,75048	0,17321
(1636)	0,94868	-0,47434	0,15811
(203)	-0,64327	-0,63246	0,15811
(746)	-0,36893	-0,47434	0,15811
Fatty acid (611)	-0,63246	-0,63246	0,15811
Fatty acid (767)	-0,47434	-0,47434	0,15811
Stearic acid (605)	-0,47434	-0,47434	0,15811
(2073)	0,34641	-0,43301	0,072169
Small peptide (1650)	0,94868	-0,36893	0,052705
(1048)	0,86603	0	0
(1076)	0,86603	0	0
(1100)	0,9083	0	0
(1121)	0,9649	0,48245	0
(1303)	0,9649	0	0
(1305)	0,9649	0	0
(1393)	0,9649	0,75048	0
(1569)	0,28868	0	0
(1767)	0,28868	0	0
(1999)	0,48245	0	0
(2104)	0,17321	0	0
(2119)	0,86603	0	0
(2138)	0,48245	0,33029	0
(2149)	0,33029	0	0
(2R)-beta,beta-Caroten-2-ol (699)	0,86603	0,28868	0
(399)	0	0	0
(428)	0	0	0
(450)	0	0	0
(460)	0	0	0
(477)	0	0	0
(722)	0	0	0
12-hydroxyjasmonic acid related (730)	0,9649	0,48245	0

**Compound**	**HexFr**	**EtAcFr**	**ButFr**
12-hydroxyjasmonic acid related (735)	0,9649	0,48245	0
1-Monolinoleoyl-rac-glycerol related (2051)	0,9083	0	0
Amino acid related (1346)	0,9649	0,17321	0
Benzylpenicillin related (480)	0	0	0
Diethylamino hydroxybenzoyl hexyl benzoate (1401)	0,9649	0	0
Endocannabinoid (1372)	0,9649	0,48245	0
Fatty alcohol (1272)	0,48245	0	0
Fatty alcohol (1626)	0	0	0
Fatty amide (1814)	0,48245	0	0
Oleanolic acid (1323)	0,9649	0,33029	0
Oligopeptide (464)	0	0	0
Oligopeptide (476)	0	0	0
Oligopeptide (723)	0	0	0
Pheophorbide A related (2102)	0,9649	0	0
Pheophorbide A related (2108)	0,9649	0,48245	0
Pheophorbide A related (2128)	0,48245	0,48245	0
Phtalate contaminant (1049)	0,9649	0	0
Pyrazine/Piperazine alkaloid (135)	0	0	0
Terpenoid (1474)	0,9649	0	0
(1659)	0,94868	-0,47434	-0,15811
(733)	0,15811	-0,48245	-0,15811
Cyclamide (280)	-0,48245	-0,47434	-0,15811
(580)	0,47633	-0,17321	-0,17321
(614)	-0,63246	-0,63246	-0,21082
Sphingoid base (680)	-0,47434	-0,47434	-0,26352
(148)	-0,58966	-0,57975	-0,31623
(1890)	-0,62622	-0,46188	-0,33029
(658)	-0,33029	-0,33029	-0,33029
(579)	0,47434	-0,36893	-0,36893
(1498)	0,73786	-0,47434	-0,47434
(1566)	-0,47434	-0,47434	-0,47434
(531)	0,47434	-0,47434	-0,47434

Compound	HexFr	EtAcFr	ButFr
(631)	-0,47434	-0,47434	-0,47434
(634)	-0,47434	-0,47434	-0,47434
(637)	-0,47434	-0,47434	-0,47434
(644)	-0,26352	-0,47434	-0,47434
(646)	-0,26352	-0,47434	-0,47434
(648)	-0,26352	-0,47434	-0,47434
Carbendazim (233)	-0,47434	-0,47434	-0,47434
Monoacylglicerol (683)	-0,47434	-0,47434	-0,47434
Pyraclostrobin (760)	-0,47434	-0,47434	-0,47434
Pyraclostrobin (758)	-0,05271	-0,47434	-0,47434
(505)	-0,47633	-0,48245	-0,48245
(555)	-0,94868	-0,94868	-0,52705
p-coumaric acid [M-H2O+H] (236)	-0,53606	-0,52705	-0,52705
(64)	-0,57801	-0,57801	-0,57801
(1567)	-0,57975	-0,57975	-0,57975
(572)	0,84327	-0,57975	-0,57975
(708)	-0,94868	-0,94868	-0,63246
Amine (630)	-0,63246	-0,63246	-0,63246
(2052)	0,63246	-0,10585	-0,66058
p-Coumaric acid (124)	-0,75048	-0,73786	-0,68516
(596)	-0,73786	-0,73786	-0,73786
Fatty acid conjugate (529)	-0,73786	-0,73786	-0,73786
Guanosine (141)	-0,85769	-0,84327	-0,73786
(65)	-0,9649	-0,79057	-0,79057
(74)	-0,80408	-0,79057	-0,79057
Amino acid related (68)	-0,80408	-0,79057	-0,79057
Primary amide (140)	-0,9649	-0,94868	-0,79057
Fatty alcohol (1919)	-0,82035	-0,73786	-0,82035
(267)	-0,85769	-0,84327	-0,84327
Amino acid related (70)	-0,85769	-0,84327	-0,84327
Oligopeptide (1600)	-0,84327	-0,84327	-0,84327
Sphingoid base (543)	-0,84327	-0,84327	-0,84327
(59)	-0,89598	-0,89598	-0,89598

Compound	HexFr	EtAcFr	ButFr
(115)	-0,9649	-0,94868	-0,94868
(1596)	-0,94868	-0,94868	-0,94868
(21)	-0,94868	-0,94868	-0,94868
(27)	-0,9649	-0,95266	-0,94868
(36)	-0,9649	-0,9649	-0,94868
(537)	-0,94868	-0,94868	-0,94868
(546)	-0,94868	-0,94868	-0,94868
(63)	-0,9649	-0,94868	-0,94868
(654)	-0,94868	-0,94868	-0,94868
(666)	-0,94868	-0,94868	-0,94868
(667)	-0,94868	-0,94868	-0,94868
(673)	-0,94868	-0,94868	-0,94868
(700)	-0,94868	-0,94868	-0,94868
(71)	-0,9649	-0,95266	-0,94868
(711)	-0,94868	-0,94868	-0,94868
(82)	-0,9649	-0,94868	-0,94868
3-p-coumaroylquinic acid related (242)	-0,9649	-0,9649	-0,94868
Anthranilate (76)	-0,94868	-0,94868	-0,94868
Carbohydrate conjugate (58)	-0,9649	-0,94868	-0,94868
L-arginine (55)	-0,9649	-0,94868	-0,94868
L-histidine (52)	-0,9649	-0,94868	-0,94868
Monoacylglycerol (550)	-0,94868	-0,94868	-0,94868
Monoacylglycerol (562)	-0,94868	-0,94868	-0,94868
Organosulfonic acid derivative (29)	-0,9649	-0,94868	-0,94868
Secoiridoid monoterpenoid (1599)	-0,94868	-0,94868	-0,94868
Sphingoid base (538)	-0,94868	-0,94868	-0,94868
Amino acid related (80)	-0,9649	-0,9649	-0,95266

HexFr: hexane fraction of CvExt. EtAcFr: ethyl acetate fraction of CvExt. ButFr: butanolic fraction of CvExt. Full LC-MS/MS dataset can be checked at https://massive.ucsd.edu/ProteoSAFe/dataset.jsp?accession=MSV000099903.

Supplementary Table S4: Complete Spearman correlation analysis of anti-AGE activity in the most active fractions of fractions of *Cissus verticillata* leaf hydroethanolic extract (CvExt) in negative mode (ESI^-).

Compound	HexFr	EtAcFr	ButFr
(144)	0	0,9649	0,9649
(171)	0	0,9649	0,9649
(185)	0	0,9649	0,9649
(188)	0	0,9649	0,9649
(201)	0	0,9649	0,9649
(208)	0	0,9649	0,9649
(214)	0	0,9649	0,9649
(216)	0,23094	0,9649	0,9649
(220)	0	0,9649	0,9649
(222)	0	0,9649	0,9649
(233)	0	0,9649	0,9649
(235)	0,48245	0,9649	0,9649
(241)	0	0,9649	0,9649
(243)	0	0,9649	0,9649
(244)	0	0,9649	0,9649
(245)	0	0,9649	0,9649
(249)	0	0,9649	0,9649
(260)	0	0,9649	0,9649
(261)	0	0,9649	0,9649
(263)	0	0,9649	0,9649
(265)	0,48245	0,9649	0,9649
(266)	0	0,9649	0,9649
(267)	0	0,9649	0,9649
(268)	0	0,9649	0,9649
(270)	0	0,9649	0,9649
(272)	0	0,9649	0,9649
(273)	0	0,9649	0,9649
(274)	0,58966	0,9649	0,9649

**Compound**	**HexFr**	**EtAcFr**	**ButFr**
(276)	0	0,9649	0,9649
(281)	0	0,9649	0,9649
(282)	0	0,9649	0,9649
(283)	0	0,9649	0,9649
(285)	0	0,9649	0,9649
(288)	0	0,9649	0,9649
(289)	0	0,9649	0,9649
(290)	0	0,9649	0,9649
(293)	0	0,9649	0,9649
(294)	0	0,9649	0,9649
(296)	0	0,9649	0,9649
(298)	0	0,9649	0,9649
(299)	0,48245	0,9649	0,9649
(301)	0	0,9649	0,9649
(302)	0	0,9649	0,9649
(303)	0	0,9649	0,9649
(304)	0	0,9649	0,9649
(306)	0	0,9649	0,9649
(307)	0	0,9649	0,9649
(308)	0	0,9649	0,9649
(310)	0	0,9649	0,9649
(314)	0	0,9649	0,9649
(315)	0	0,9649	0,9649
(319)	0	0,9649	0,9649
(320)	0	0,9649	0,9649
(322)	0	0,9649	0,9649
(323)	0,48245	0,9649	0,9649
(325)	0	0,9649	0,9649
(326)	0	0,9649	0,9649
(327)	0	0,9649	0,9649
(329)	0	0,9649	0,9649
(330)	0	0,9649	0,9649
(331)	0	0,9649	0,9649

**Compound**	**HexFr**	**EtAcFr**	**ButFr**
(334)	0	0,9649	0,9649
(337)	0	0,9649	0,9649
(339)	0	0,9649	0,9649
(341)	0	0,9649	0,9649
(344)	0	0,9649	0,9649
(346)	0	0,9649	0,9649
(348)	0,9649	0,9649	0,9649
(349)	0	0,9649	0,9649
(350)	0,17321	0,9649	0,9649
(353)	0	0,9649	0,9649
(362)	0	0,9649	0,9649
(363)	0	0,9649	0,9649
(381)	0	0,9649	0,9649
(394)	0	0,23094	0,9649
(413)	0	0,58966	0,9649
(420)	0	0,9113	0,9649
(424)	0	0,33029	0,9649
(425)	0,9649	0,9649	0,9649
(433)	0	0	0,9649
(451)	0,48245	0,9649	0,9649
(461)	0	0,23094	0,9649
(488)	0,48245	0,9649	0,9649
(493)	0,48245	0,9649	0,9649
(504)	0,48245	0,9649	0,9649
(513)	0,48245	0,9649	0,9649
(521)	0,48245	0,48245	0,9649
(555)	0	0	0,9649
(563)	0	0,48245	0,9649
(572)	0	0	0,9649
(581)	0,9649	0,64327	0,9649
2-({6-O-[(2E)-3-(4-Hydroxyphenyl)-2-propenoyl]-beta-D-glucopyranosyl}oxy)-3-phenylacrylic acid (277)	0	0,9649	0,9649

**Compound**	**HexFr**	**EtAcFr**	**ButFr**
2-(3,4-dihydroxyphenyl)-5,8-dihydroxy-7-methoxy-3-[(2S,3R,4R,5R,6S)-3,4,5-trihydroxy-6-methyloxan-2-yl]oxychromen-4-one (231)	0	0,9649	0,9649
4-oxododecanedioic acid (252)	0,44039	0,9649	0,9649
7-(geranyloxy)coumarin (450)	0	0,17321	0,9649
9,12,13,TriHODE related (376)	0	0,9649	0,9649
Afzelin (255)	0	0,9649	0,9649
Afzelin (256)	0	0,9649	0,9649
Afzelin related (262)	0	0,9649	0,9649
Coumaric acid (212)	0	0,9649	0,9649
Glc-Glc-octadecatrienoyl-sn-glycerol (431)	0,48245	0,9649	0,9649
Glc-Glc-octadecatrienoyl-sn-glycerol (441)	0,48245	0,9649	0,9649
Kaji-ichigoside F1 (352)	0	0,9649	0,9649
Myricetin 3-O-rutinoside (152)	0	0,9649	0,9649
Myricetin-3-O-galactoside (164)	0	0,9649	0,9649
Myricitrin (189)	0	0,9649	0,9649
Myricitrin (278)	0	0,9649	0,9649
Myricitrin (284)	0	0,9649	0,9649
PI(18:1/0:0) (444)	0	0,48245	0,9649
Quercitrin (228)	0	0,9649	0,9649
(150)	-0,17321	0,74096	0,95266
(226)	-0,17321	0,95266	0,95266
(250)	-0,17321	0,95266	0,95266
(251)	-0,17321	0,95266	0,95266
(279)	-0,17321	0,95266	0,95266
(342)	-0,17321	0,95266	0,95266
(462)	0,47633	0,47633	0,95266
3-Hydroxycinnamic acid (197)	-0,17321	0,95266	0,95266
Chlorogenic acid (159)	-0,17321	0,95266	0,95266
(10)	0,63246	0,94868	0,94868
(101)	-0,48245	0,94868	0,94868
(105)	-0,48245	0,94868	0,94868
(106)	-0,48245	0,94868	0,94868

Compound	HexFr	EtAcFr	ButFr
(108)	-0,48245	0,94868	0,94868
(109)	-0,48245	0,94868	0,94868
(11)	0,94868	0,94868	0,94868
(124)	-0,48245	0,57975	0,94868
(129)	-0,48245	0,94868	0,94868
(130)	-0,48245	0,94868	0,94868
(132)	-0,48245	0,94868	0,94868
(134)	-0,48245	0,94868	0,94868
(135)	-0,48245	0,94868	0,94868
(136)	-0,48245	0,94868	0,94868
(140)	-0,48245	0,89598	0,94868
(143)	-0,48245	0,94868	0,94868
(147)	-0,48245	0,94868	0,94868
(156)	-0,48245	0,94868	0,94868
(168)	-0,48245	0,94868	0,94868
(173)	-0,48245	0,94868	0,94868
(175)	-0,48245	0,94868	0,94868
(181)	0,052705	0,94868	0,94868
(186)	-0,48245	0,94868	0,94868
(190)	-0,48245	0,94868	0,94868
(193)	-0,48245	0,94868	0,94868
(194)	-0,48245	0,94868	0,94868
(195)	-0,48245	0,94868	0,94868
(196)	-0,33029	0,94868	0,94868
(198)	-0,48245	0,94868	0,94868
(206)	-0,48245	0,052705	0,94868
(207)	-0,48245	0,94868	0,94868
(209)	-0,33029	0,94868	0,94868
(210)	-0,33029	0,94868	0,94868
(211)	-0,48245	0,89598	0,94868
(213)	-0,48245	0,94868	0,94868
(217)	-0,48245	0,94868	0,94868
(223)	-0,48245	-0,47434	0,94868

**Compound**	**HexFr**	**EtAcFr**	**ButFr**
(224)	-0,48245	0,94868	0,94868
(229)	-0,48245	0,94868	0,94868
(230)	-0,48245	0,94868	0,94868
(232)	-0,48245	0,94868	0,94868
(234)	-0,48245	0,94868	0,94868
(236)	-0,48245	0,94868	0,94868
(238)	-0,48245	0,94868	0,94868
(239)	-0,48245	0,94868	0,94868
(253)	-0,48245	0,94868	0,94868
(259)	-0,48245	0,94868	0,94868
(280)	-0,48245	0,57975	0,94868
(30)	-0,48245	0,94868	0,94868
(305)	-0,48245	0,52705	0,94868
(309)	-0,33029	0,94868	0,94868
(312)	-0,48245	0,68516	0,94868
(321)	-0,48245	0,84327	0,94868
(35)	-0,48245	0,47434	0,94868
(43)	-0,48245	0,94868	0,94868
(455)	-0,2117	0,47434	0,94868
(465)	0,94868	0,94868	0,94868
(467)	0,47434	0,47434	0,94868
(478)	-0,05271	0,47434	0,94868
(494)	-0,47434	0,47434	0,94868
(502)	-0,47434	0,47434	0,94868
(510)	0,47434	0,94868	0,94868
(550)	-0,36893	0,47434	0,94868
(570)	-0,15811	0,47434	0,94868
(571)	-0,05271	0,47434	0,94868
(68)	-0,48245	0,94868	0,94868
(80)	-0,33029	0,94868	0,94868
(81)	-0,48245	0,94868	0,94868
(82)	-0,48245	0,94868	0,94868
(85)	-0,48245	0,94868	0,94868

Compound	HexFr	EtAcFr	ButFr
(88)	-0,48245	0,94868	0,94868
(92)	-0,48245	0,94868	0,94868
(99)	-0,48245	0,94868	0,94868
1-O-(4-Coumaroyl)-beta-D-glucose (104)	-0,48245	0,94868	0,94868
6,7-Dihydroxycoumarin (139)	-0,48245	0,94868	0,94868
Afzelin (257)	-0,48245	0,94868	0,94868
Afzelin (258)	-0,47434	0,94868	0,94868
Apigenin_Hex_Hex (121)	-0,48245	0,89598	0,94868
Apigenin_Pen_Hex (148)	-0,48245	0,94868	0,94868
Apigenin_Pen_Hex (158)	-0,48245	0,89598	0,94868
Chlorogenic acid (133)	-0,48245	0,94868	0,94868
Chlorogenic acid (98)	-0,48245	0,94868	0,94868
Chlorogenic acid related (145)	-0,47434	0,94868	0,94868
Chlorogenic acid related (177)	-0,47633	0,94868	0,94868
Corchoionoside C (179)	-0,47434	0,94868	0,94868
Coumaric acid (103)	-0,48245	0,94868	0,94868
Cyanidin-3-O-rutinoside (205)	-0,48245	0,94868	0,94868
FA 18:2+3O (360)	0,47434	0,94868	0,94868
Gentisate (114)	-0,48245	0,94868	0,94868
Isochlorogenic acid B related (94)	-0,48245	0,94868	0,94868
Kinic acid (146)	-0,48245	0,94868	0,94868
Kinic acid (176)	-0,48245	0,94868	0,94868
Kinic acid (97)	-0,48245	0,94868	0,94868
Myricitrin (191)	-0,47434	0,94868	0,94868
Myricitrin (192)	-0,48245	0,94868	0,94868
Pantothenic acid-B5 (64)	-0,48245	0,94868	0,94868
Quercitrin (225)	-0,33029	0,94868	0,94868
Quercitrin (227)	-0,47434	0,94868	0,94868
Rutin (182)	-0,48245	0,94868	0,94868
Tryptophan (79)	-0,48245	0,94868	0,94868
(345)	0	0,9083	0,9083
(470)	0	0	0,9083
(110)	-0,48245	0,15811	0,89598

Compound	HexFr	EtAcFr	ButFr
(115)	-0,53606	0,84327	0,89598
(155)	-0,53606	-0,52705	0,89598
(514)	0,47434	0,47434	0,89598
(559)	0,052705	0,47434	0,89598
(560)	-0,52705	-0,31623	0,89598
(566)	0,94868	0,47434	0,89598
(358)	0	0,86603	0,86603
(355)	0,48245	0,9649	0,85769
(446)	0	0,58966	0,85769
(335)	0	0,9083	0,85325
Taxifolin (219)	0	0,9083	0,85325
(183)	-0,28868	0,84681	0,84681
(240)	0	0,8454	0,8454
(340)	0	0,8454	0,8454
(377)	0	0,8454	0,8454
(469)	0	0	0,8454
(519)	0	0	0,8454
7-(geranyloxy)coumarin (459)	0	0	0,8454
Decylbenzenesulfonic acid (468)	0	0	0,8454
Decylbenzenesulfonic acid (476)	0	0	0,8454
(41)	-0,48245	-0,47434	0,84327
(440)	0,052705	0,47434	0,84327
(51)	-0,58966	0,84327	0,84327
(62)	-0,58966	0,26352	0,84327
(95)	-0,58966	0,79057	0,84327
(567)	0,17321	0,48245	0,80408
Coumaric acid (184)	0	0,9649	0,80408
(392)	0,21918	0,9083	0,7982
(449)	0	0,21918	0,7982
(151)	-0,48245	0,79057	0,79057
(153)	-0,48245	0,79057	0,79057
(157)	-0,64327	0,79057	0,79057
(23)	0,57975	0,79057	0,79057

Compound	HexFr	EtAcFr	ButFr
(583)	-0,57801	0,57975	0,79057
(84)	-0,64327	0,79057	0,79057
Neochlorogenic acid (74)	-0,64327	0,79057	0,79057
(516)	0,28868	0,28868	0,75056
(125)	-0,69687	0,68516	0,73786
(174)	-0,69687	0,73786	0,73786
(523)	0,47434	0,47434	0,73786
3-O-Feruloylquinic acid (163)	-0,69687	0,73786	0,73786
(379)	0,58966	0,9649	0,69687
(383)	0	0,9649	0,69687
(435)	0,9649	0,9649	0,69687
(545)	0	0	0,69282
(534)	0,26352	0,47434	0,68516
Gentisate (72)	-0,60553	0,68516	0,68516
(402)	0,8454	0	0,64952
(557)	0	0	0,64952
(417)	0,9649	0,9649	0,64327
(117)	-0,80408	-0,79057	0,63246
(169)	-0,80408	0,63246	0,63246
(170)	-0,80408	-0,63246	0,63246
(200)	-0,80408	-0,79057	0,63246
(530)	0,47434	0,47434	0,63246
Ethiprole related (482)	0,94868	0,84327	0,63246
Fipronil sulfone (499)	0,94868	0,84327	0,63246
Uracil arabinoside (47)	-0,80408	0,63246	0,63246
(387)	0,9649	0,9649	0,58966
(154)	-0,69687	-0,68516	0,57975
(540)	0,36893	0,47434	0,57975
(374)	0	0,86603	0,5636
(490)	0,9649	0,69687	0,53606
(165)	-0,9113	-0,89598	0,52705
(178)	-0,9113	-0,89598	0,52705
(399)	0	0,48245	0,48245

**Compound**	**HexFr**	**EtAcFr**	**ButFr**
(400)	0,9649	0,9649	0,48245
(423)	0,9649	0,9649	0,48245
(429)	0,9649	0,9649	0,48245
(437)	0,9649	0,9649	0,48245
(439)	0,9649	0,9649	0,48245
(472)	0,9649	0,9649	0,48245
(474)	0,9649	0,9649	0,48245
(480)	0,9649	0,9649	0,48245
(492)	0,9649	0,9649	0,48245
(509)	0,9649	0,9649	0,48245
(568)	0,48245	0,9649	0,48245
(579)	0,9649	0,9649	0,48245
(582)	0,9649	0,80408	0,48245
Dihydrokaempferol (271)	0	0,9649	0,48245
(118)	-0,9649	0,47434	0,47434
(123)	-0,33029	0,47434	0,47434
(127)	-0,48245	0,47434	0,47434
(131)	-0,9649	-0,94868	0,47434
(149)	-0,94868	-0,94868	0,47434
(48)	-0,9649	0,47434	0,47434
(508)	0,9083	0	0,46188
(52)	-0,9649	-0,94868	0,36893
(414)	0,9649	0,9649	0,33029
(122)	-0,75048	-0,73786	0,31623
(443)	0,9649	0,9649	0,28868
5-trans-Prostaglandin D2 related (406)	0,9649	0,9649	0,28868
(16)	-0,52705	-0,52705	0,26352
(49)	-0,9649	-0,94868	0,26352
(552)	0,9649	0,9113	0,23094
(405)	0,9649	0,9649	0,17321
(479)	0,9649	0,9649	0,17321
(517)	0,9649	0,9649	0,17321
(564)	0,9649	0,9649	0,17321

Compound	HexFr	EtAcFr	ButFr
(128)	-0,9649	-0,42164	0,052705
(160)	-0,85769	-0,84327	0,052705
(161)	-0,9649	-0,94868	0
(365)	0,9083	0,9083	0
(373)	0,9649	0	0
(395)	0,86603	0,86603	0
(404)	0,86603	0,86603	0
(428)	0,9083	0,9083	0
(434)	0,9649	0,80408	0
(445)	0,9649	0,9649	0
(454)	0,9649	0,9649	0
(471)	0,9649	0,9649	0
(473)	0,9649	0,9649	0
(485)	0,9649	0,9649	0
(491)	0,9083	0,7982	0
(495)	0,9649	0,9649	0
(496)	0,9649	0,9649	0
(505)	0,9083	0,9083	0
(518)	0,9649	0,9649	0
(520)	0,9649	0,9649	0
(526)	0,9649	0,48245	0
(527)	0,9649	0,48245	0
(536)	0,9649	0	0
(537)	0,9649	0	0
(539)	0,9649	0	0
(542)	0,9649	0,9649	0
(543)	0,9649	0,9649	0
(544)	0,9083	0,9083	0
(549)	0,9649	0,48245	0
(551)	0,86603	0,62622	0
(580)	0,9649	0,9649	0
Echinocystic acid (561)	0,9649	0,9649	0

Compound	HexFr	EtAcFr	ButFr
methyl (2E,4E,8E)-7,13-dihydroxy-4,8,12-trimethyltetradeca-2,4,8-trienoate (475)	0,9649	0,9113	0
methyl (2E,4E,8E)-7,13-dihydroxy-4,8,12-trimethyltetradeca-2,4,8-trienoate (483)	0,9083	0,9083	0
(533)	0,69282	-0,43301	-0,07217
(86)	-0,94868	-0,94868	-0,15811
(142)	-0,85769	-0,26352	-0,26352
(17)	-0,47434	-0,47434	-0,26352
(535)	0,47434	0,47434	-0,26352
(573)	0,16082	0,48245	-0,26803
(448)	0,94868	-0,48245	-0,48245
(541)	0,15811	0,47434	-0,52705
(77)	-0,9649	-0,94868	-0,57975
(46)	-0,94868	-0,94868	-0,68516
(111)	-0,80408	-0,79057	-0,79057
Homoaspartic acid (12)	-0,94868	-0,79057	-0,79057
(67)	-0,85769	-0,84327	-0,84327
Coumaroyl + C6H9O8 (116)	-0,85769	-0,84327	-0,84327
(102)	-0,9649	-0,94868	-0,89598
(119)	-0,9649	-0,94868	-0,89598
(65)	-0,9113	-0,89598	-0,89598
(89)	-0,9113	-0,89598	-0,89598
(1)	-0,94868	-0,94868	-0,94868
(13)	-0,95266	-0,94868	-0,94868
(14)	-0,95266	-0,94868	-0,94868
(15)	-0,94868	-0,94868	-0,94868
(18)	-0,9649	-0,94868	-0,94868
(19)	-0,94868	-0,94868	-0,94868
(2)	-0,9649	-0,9649	-0,94868
(20)	-0,95266	-0,94868	-0,94868
(22)	-0,9649	-0,94868	-0,94868
(24)	-0,9649	-0,94868	-0,94868
(25)	-0,9649	-0,94868	-0,94868

Compound	HexFr	EtAcFr	ButFr
(27)	-0,94868	-0,94868	-0,94868
(28)	-0,9649	-0,94868	-0,94868
(29)	-0,9649	-0,94868	-0,94868
(3)	-0,94868	-0,94868	-0,94868
(33)	-0,9649	-0,94868	-0,94868
(34)	-0,9649	-0,94868	-0,94868
(36)	-0,9649	-0,94868	-0,94868
(38)	-0,9649	-0,94868	-0,94868
(4)	-0,95266	-0,94868	-0,94868
(5)	-0,94868	-0,94868	-0,94868
(57)	-0,9649	-0,95266	-0,94868
(6)	-0,9649	-0,94868	-0,94868
(60)	-0,9649	-0,94868	-0,94868
(7)	-0,9649	-0,94868	-0,94868
(73)	-0,9649	-0,94868	-0,94868
(8)	-0,9649	-0,94868	-0,94868
(91)	-0,9649	-0,94868	-0,94868
Benzoic acid + 2O_ O-Hex (63)	-0,9649	-0,94868	-0,94868
Citric acid (45)	-0,9649	-0,94868	-0,94868
Coumaroyl + C6H9O8 (78)	-0,9649	-0,94868	-0,94868
Coumaroyl + C6H9O8 (90)	-0,9649	-0,94868	-0,94868
Homoarginin (9)	-0,9649	-0,94868	-0,94868
(21)	-0,9649	-0,94868	-0,95266
(26)	-0,9649	-0,94868	-0,95266
Citric acid (39)	-0,9649	-0,95266	-0,9649

HexFr: hexane fraction of CvExt. EtAcFr: ethyl acetate fraction of CvExt. ButFr: butanolic fraction of CvExt. Full LC-MS/MS dataset can be checked at https://massive.ucsd.edu/ProteoSAFe/dataset.jsp?accession=MSV000099903.

Supplementary Table S5: Complete Spearman correlation analysis of anti-PCO activity in the most active fractions of *Cissus verticillata* leaf hydroethanolic extract (CvExt) in positive mode (ESI^+).

Compound	EtAcFr	ButFr	HEtFr
Cyclamide (280)	0,9649	0,9649	0,9649
Amino acid related (268)	0,9649	0,9649	0,9649
Peptide alkaloid (265)	0,9649	0,9649	0,9649
Pyridine alkaloid (159)	0,9649	0,9649	0,9649
(313)	0,9649	0,9649	0,9649
p-coumaric acid [M-H2O+H] (236)	0,9113	0,9649	0,9113
Amino acid related (418)	0,8454	0,9649	0,8454
Oligopeptide (422)	0,8454	0,9649	0,8454
Oligopeptide (416)	0,8454	0,9649	0,8454
Oligopeptide (417)	0,8454	0,9649	0,8454
(259)	0,8454	0,9649	0,8454
(412)	0,8454	0,9649	0,8454
(441)	0,8454	0,9649	0,8454
Amino acid related (68)	0,64327	0,9649	0,64327
(74)	0,64327	0,9649	0,64327
Amino acid related (70)	0,58966	0,9649	0,58966
(267)	0,58966	0,9649	0,58966
L-arginine (55)	0,48245	0,9649	0,48245
L-histidine (52)	0,48245	0,9649	0,48245
Carbohydrate conjugate (58)	0,48245	0,9649	0,48245
3-p-coumaroylquinic acid related (242)	0,48245	0,9649	0,48245
Organosulfonic acid derivative (29)	0,48245	0,9649	0,48245
(27)	0,48245	0,9649	0,48245
(63)	0,48245	0,9649	0,48245
(71)	0,48245	0,9649	0,48245
(82)	0,48245	0,9649	0,48245
(115)	0,48245	0,9649	0,48245
Oligopeptide (408)	0,69282	0,95266	0,69282

**Compound**	**EtAcFr**	**ButFr**	**HEtFr**
(1887)	0,69282	0,95266	0,69282
Fatty acid (642)	0,94868	0,94868	0,94868
Fatty acid (767)	0,94868	0,94868	0,94868
Monoacylglicerol (683)	0,94868	0,94868	0,94868
Carbendazim (233)	0,94868	0,94868	0,94868
Sphingoid base (680)	0,94868	0,94868	0,94868
Stearic acid (605)	0,94868	0,94868	0,94868
Pyraclostrobin (760)	0,94868	0,94868	0,94868
(678)	0,94868	0,94868	0,94868
(746)	0,89598	0,94868	0,89598
(766)	0,89598	0,94868	0,89598
(614)	0,79057	0,94868	0,79057
Fatty acid conjugate (529)	0,68516	0,94868	0,68516
Pheophorbide A related (1483)	0,57801	0,94868	0,57801
(490)	0,57801	0,94868	0,57801
(59)	0,52705	0,94868	0,52705
Pheophorbide A related (1322)	0,48245	0,94868	0,48245
(465)	0,48245	0,94868	0,48245
(493)	0,48245	0,94868	0,48245
Anthranilate (76)	0,47434	0,94868	0,47434
Monoacylglycerol (550)	0,47434	0,94868	0,47434
Monoacylglycerol (562)	0,47434	0,94868	0,47434
Sphingoid base (764)	0,47434	0,94868	0,47434
Sphingoid base (538)	0,47434	0,94868	0,47434
Sphingoid base (609)	0,47434	0,94868	0,47434
Secoiridoid monoterpenoid (1599)	0,47434	0,94868	0,47434
(21)	0,47434	0,94868	0,47434
(537)	0,47434	0,94868	0,47434
(546)	0,47434	0,94868	0,47434
(2037)	0,47434	0,94868	0,47434
(148)	0,85769	0,9113	0,85769
(402)	0,8454	0,9113	0,8454
p-Coumaric acid (124)	0,69687	0,9113	0,69687

Compound	EtAcFr	ButFr	HEtFr
(36)	0,48245	0,9083	0,48245
(596)	0,68516	0,89598	0,68516
(1262)	0,57975	0,89598	0,57975
(396)	0,8454	0,86603	0,8454
Amino acid related (80)	0,48245	0,86603	0,48245
Guanosine (141)	0,58966	0,85769	0,58966
(658)	0,9083	0,8454	0,9083
Fatty alcohol (1626)	0,8454	0,8454	0,8454
Oligopeptide (464)	0,8454	0,8454	0,8454
Oligopeptide (476)	0,8454	0,8454	0,8454
Oligopeptide (723)	0,8454	0,8454	0,8454
Benzylpenicillin related (480)	0,8454	0,8454	0,8454
Pyrazine/Piperazine alkaloid (135)	0,8454	0,8454	0,8454
(399)	0,8454	0,8454	0,8454
(428)	0,8454	0,8454	0,8454
(450)	0,8454	0,8454	0,8454
(460)	0,8454	0,8454	0,8454
(477)	0,8454	0,8454	0,8454
(722)	0,8454	0,8454	0,8454
Oligopeptide (1600)	0,57975	0,84327	0,57975
Fatty acid conjugate (584)	0,48245	0,84327	0,48245
(1596)	0,47434	0,84327	0,47434
(654)	0,47434	0,84327	0,47434
(666)	0,47434	0,84327	0,47434
(203)	0,80408	0,80408	0,80408
Primary amide (140)	0,48245	0,80408	0,48245
(65)	0,48245	0,80408	0,48245
Cyclamide (1167)	0,95266	0,79388	0,95266
Dibutoxyethoxyethyl adipate related (786)	0,94868	0,79057	0,94868
(1566)	0,94868	0,79057	0,94868
Fatty acid (611)	0,79057	0,79057	0,79057
(667)	0,47434	0,79057	0,47434
L-phenylalanine (175)	0,9649	0,75048	0,9649

Compound	EtAcFr	ButFr	HEtFr
L-tryptophan (213)	0,9649	0,75048	0,9649
L-tryptophan (214)	0,9649	0,75048	0,9649
Indole-3-carboxaldehyde (218)	0,9649	0,75048	0,9649
(85)	0,9649	0,75048	0,9649
(356)	0,69687	0,73786	0,69687
L-phenylalanine (176)	0,9649	0,69687	0,9649
L-phenylalanine (174)	0,9649	0,69687	0,9649
Kaempferol O-rhamnoside (430)	0,9649	0,69687	0,9649
(188)	0,9649	0,69687	0,9649
(707)	0,86603	0,69687	0,86603
(505)	0,95266	0,69282	0,95266
(2104)	0,69282	0,69282	0,69282
(687)	0,94868	0,68516	0,94868
(2007)	0,48245	0,68516	0,48245
(2107)	0,47434	0,68516	0,47434
(64)	0,66058	0,64952	0,66058
Kaempferol O-rhamnoside (429)	0,9649	0,64327	0,9649
(2100)	0,9649	0,64327	0,9649
Amino acid related (293)	0,86603	0,64327	0,86603
a-aminoacid derivative (295)	0,8454	0,64327	0,8454
(364)	0,77067	0,63511	0,77067
Oligopeptide (317)	0,71563	0,63511	0,71563
(1567)	0,84327	0,63246	0,84327
(2005)	0,48245	0,63246	0,48245
(673)	0,47434	0,63246	0,47434
(708)	0,47434	0,63246	0,47434
Neochlorogenic acid (239)	0,9649	0,58966	0,9649
Purine nucleoside (691)	0,94868	0,57975	0,94868
(1179)	0,89598	0,57975	0,89598
(733)	0,63246	0,57975	0,63246
4-acetylbutyric-acid_1-octacosanol (1744)	0,47434	0,57975	0,47434
(700)	0,47434	0,57975	0,47434
(711)	0,47434	0,57975	0,47434

Compound	EtAcFr	ButFr	HEtFr
(1273)	0,47434	0,57975	0,47434
(2149)	0,57801	0,57801	0,57801
Purine alkaloid (231)	0,94868	0,52705	0,94868
Pheophorbide A related (2091)	0,48245	0,52705	0,48245
(555)	0,47434	0,52705	0,47434
1H-pyrrolo[3,2-b]pyridine-5-carboxylic acid (241)	0,9649	0,48245	0,9649
Alkaloid (258)	0,9649	0,48245	0,9649
Tryptophan-related (256)	0,9649	0,48245	0,9649
Val-Leu (227)	0,9649	0,48245	0,9649
3-p-coumaroylquinic acid (287)	0,9649	0,48245	0,9649
Fatty acid (626)	0,9649	0,48245	0,9649
Myricetin-3-Xyloside (344)	0,9649	0,48245	0,9649
Myricetin-3-Xyloside (345)	0,9649	0,48245	0,9649
Quercitrin (386)	0,9649	0,48245	0,9649
Oligopeptide (395)	0,9649	0,48245	0,9649
Bis-(2-ehtylhexyl) phthalate related (312)	0,9649	0,48245	0,9649
Bis-(2-ehtylhexyl) phthalate related (333)	0,9649	0,48245	0,9649
Adenin (84)	0,9649	0,48245	0,9649
Adenin (110)	0,9649	0,48245	0,9649
Unsaturated fatty acid (623)	0,9649	0,48245	0,9649
Corymboside (282)	0,9649	0,48245	0,9649
Isoshaftoside (305)	0,9649	0,48245	0,9649
p-coumaric acid (289)	0,9649	0,48245	0,9649
p-coumaric acid (323)	0,9649	0,48245	0,9649
Vicenin 2 (262)	0,9649	0,48245	0,9649
(244)	0,9649	0,48245	0,9649
(261)	0,9649	0,48245	0,9649
(272)	0,9649	0,48245	0,9649
(286)	0,9649	0,48245	0,9649
(324)	0,9649	0,48245	0,9649
(558)	0,9649	0,48245	0,9649
(246)	0,9113	0,48245	0,9113
Rutin (335)	0,9083	0,48245	0,9083

Compound	EtAcFr	ButFr	HEtFr
(739)	0,86603	0,48245	0,86603
Tetracosanoic-acid_1,4-cyclohexanediol (1233)	0,8454	0,48245	0,8454
PC(0:0/16:0) (748)	0,8454	0,48245	0,8454
O-glycosyl compound (303)	0,8454	0,48245	0,8454
Cyanidin-3-O-rutinoside (366)	0,8454	0,48245	0,8454
Esculetin (279)	0,8454	0,48245	0,8454
Triphenylphosphine oxide (338)	0,8454	0,48245	0,8454
(1110)	0,8454	0,48245	0,8454
Oligopeptide (314)	0,80408	0,48245	0,80408
(143)	0,80408	0,48245	0,80408
Pheophorbide A related (2128)	0,48245	0,48245	0,48245
Fatty alcohol (1272)	0,48245	0,48245	0,48245
Fatty amide (1814)	0,48245	0,48245	0,48245
Adenin (106)	0,48245	0,48245	0,48245
(1999)	0,48245	0,48245	0,48245
(2138)	0,48245	0,48245	0,48245
(580)	0,47633	0,48245	0,47633
Fatty acid (570)	0,95266	0,47633	0,95266
1-Oleoyl-sn-glycero-3-phosphoethanolamine related (734)	0,69282	0,47633	0,69282
Quercitrin (385)	0,94868	0,47434	0,94868
Unsaturated fatty acid (557)	0,94868	0,47434	0,94868
Tetradecanoic acid (496)	0,94868	0,47434	0,94868
(637)	0,94868	0,47434	0,94868
(631)	0,94868	0,47434	0,94868
(440)	0,94868	0,47434	0,94868
(634)	0,94868	0,47434	0,94868
(610)	0,94868	0,47434	0,94868
(498)	0,84327	0,47434	0,84327
(644)	0,84327	0,47434	0,84327
(646)	0,84327	0,47434	0,84327
(648)	0,84327	0,47434	0,84327
Amine (630)	0,79057	0,47434	0,79057

Compound	EtAcFr	ButFr	HEtFr
Pyraclostrobin (758)	0,73786	0,47434	0,73786
(336)	0,64327	0,47434	0,64327
Sphingoid base (543)	0,57975	0,47434	0,57975
Fatty amide (1328)	0,57801	0,47434	0,57801
Pheophorbide A (2078)	0,48245	0,47434	0,48245
Pheophorbide A related (1207)	0,48245	0,47434	0,48245
Fatty acid (2098)	0,48245	0,47434	0,48245
(1139)	0,48245	0,47434	0,48245
Pheophorbide A (1956)	0,47434	0,47434	0,47434
Fatty acid (710)	0,47434	0,47434	0,47434
Fatty acid (877)	0,47434	0,47434	0,47434
Fatty amide (1395)	0,47434	0,47434	0,47434
Citronellol (442)	0,47434	0,47434	0,47434
(531)	0,47434	0,47434	0,47434
(444)	0,47434	0,47434	0,47434
(497)	0,47434	0,47434	0,47434
(532)	0,47434	0,47434	0,47434
(579)	0,47434	0,47434	0,47434
Fatty alcohol (1919)	0,2117	0,42885	0,2117
(1890)	0,21918	0,34442	0,21918
(1578)	0,43301	0,28868	0,43301
(117)	0,17321	0,17321	0,17321
(1569)	0,072169	0,072169	0,072169
(1767)	0,072169	0,072169	0,072169
(586)	0,053606	0,052705	0,053606
(1498)	-0,05271	-0,05271	-0,05271
Carotenoid (1884)	-0,10541	-0,15811	-0,10541
(1735)	-0,43301	-0,17321	-0,43301
(453)	-0,64952	-0,33029	-0,64952
Small peptide (1650)	-0,47434	-0,47434	-0,47434
(1636)	-0,47434	-0,47434	-0,47434
(1659)	-0,47434	-0,47434	-0,47434
10S-Hydroxypheophorbide A (1697)	-0,47633	-0,47434	-0,47633

Compound	EtAcFr	ButFr	HEtFr
9-HOTrE (696)	-0,47633	-0,47434	-0,47633
10S-Hydroxypheophorbide A (1785)	-0,48245	-0,47434	-0,48245
Pheophorbide A related (1984)	-0,48245	-0,47434	-0,48245
Pheophorbide A related (1666)	-0,48245	-0,47434	-0,48245
Pheophorbide A related (1867)	-0,48245	-0,47434	-0,48245
Pheophorbide A related (1983)	-0,48245	-0,47434	-0,48245
Solvent contaminat (2148)	-0,48245	-0,47434	-0,48245
Endocannabinoid (1081)	-0,48245	-0,47434	-0,48245
Triterpenoid (1860)	-0,48245	-0,47434	-0,48245
(544)	-0,48245	-0,47434	-0,48245
(1111)	-0,48245	-0,47434	-0,48245
(2087)	-0,57801	-0,47434	-0,57801
(572)	-0,57975	-0,47434	-0,57975
Monoacylglicerol (639)	-0,48245	-0,47633	-0,48245
(705)	-0,48245	-0,47633	-0,48245
(737)	-0,48245	-0,47633	-0,48245
Amino acid related (1346)	-0,48245	-0,48245	-0,48245
Pheophorbide A related (2102)	-0,48245	-0,48245	-0,48245
Pheophorbide A related (2108)	-0,48245	-0,48245	-0,48245
Endocannabinoid (1372)	-0,48245	-0,48245	-0,48245
12-hydroxyjasmonic acid related (735)	-0,48245	-0,48245	-0,48245
12-hydroxyjasmonic acid related (730)	-0,48245	-0,48245	-0,48245
Phtalate contaminant (1049)	-0,48245	-0,48245	-0,48245
Terpenoid (1474)	-0,48245	-0,48245	-0,48245
Oleanolic acid (1323)	-0,48245	-0,48245	-0,48245
Diethylamino hydroxybenzoyl hexyl benzoate (1401)	-0,48245	-0,48245	-0,48245
(1121)	-0,48245	-0,48245	-0,48245
(1303)	-0,48245	-0,48245	-0,48245
(1305)	-0,48245	-0,48245	-0,48245
(1393)	-0,48245	-0,48245	-0,48245
(2052)	-0,79057	-0,48245	-0,79057
(452)	-0,8454	-0,48245	-0,8454
Benzenoid (421)	-0,47434	-0,52705	-0,47434

**Compound**	**EtAcFr**	**ButFr**	**HEtFr**
1-Monolinoleoyl-rac-glycerol related (2051)	-0,57801	-0,57801	-0,57801
(1100)	-0,57801	-0,57801	-0,57801
Terpenoid (1440)	-0,8454	-0,58966	-0,8454
Benzenoid (420)	-0,48245	-0,63246	-0,48245
(511)	-0,8454	-0,64327	-0,8454
(512)	-0,8454	-0,64327	-0,8454
Oleic acid [M+NH4] (1254)	-0,47434	-0,68516	-0,47434
(2R)-beta,beta-Caroten-2-ol (699)	-0,69282	-0,69282	-0,69282
(1048)	-0,69282	-0,69282	-0,69282
(1076)	-0,69282	-0,69282	-0,69282
(2073)	-0,69282	-0,69282	-0,69282
(2119)	-0,69282	-0,69282	-0,69282
(775)	-0,48245	-0,79057	-0,48245
Amine (1352)	-0,8454	-0,8454	-0,8454
L-phenylalanine related (979)	-0,8454	-0,8454	-0,8454
Pheophorbide A related (2110)	-0,8454	-0,8454	-0,8454
Wax diester (2059)	-0,8454	-0,8454	-0,8454
Oligopeptide (965)	-0,8454	-0,8454	-0,8454
Oligopeptide (2131)	-0,8454	-0,8454	-0,8454
Bis(2-ethylhexyl) phthalate (2121)	-0,8454	-0,8454	-0,8454
Dibutylphthalate (977)	-0,8454	-0,8454	-0,8454
Diacylglycerol (1915)	-0,8454	-0,8454	-0,8454
Diphenylphosphate (1329)	-0,8454	-0,8454	-0,8454
Diradylglycerol (1703)	-0,8454	-0,8454	-0,8454
Lipopeptide (2132)	-0,8454	-0,8454	-0,8454
Mefenamic acid (752)	-0,8454	-0,8454	-0,8454
(1614)	-0,8454	-0,8454	-0,8454
(598)	-0,8454	-0,8454	-0,8454
(713)	-0,8454	-0,8454	-0,8454
(715)	-0,8454	-0,8454	-0,8454
(566)	-0,8454	-0,8454	-0,8454
(608)	-0,8454	-0,8454	-0,8454
(762)	-0,8454	-0,8454	-0,8454

Compound	EtAcFr	ButFr	HEtFr
(989)	-0,8454	-0,8454	-0,8454
(1018)	-0,8454	-0,8454	-0,8454
(1047)	-0,8454	-0,8454	-0,8454
(1339)	-0,8454	-0,8454	-0,8454
(1619)	-0,8454	-0,8454	-0,8454
(1798)	-0,8454	-0,8454	-0,8454
(1870)	-0,8454	-0,8454	-0,8454
(1871)	-0,8454	-0,8454	-0,8454
(2061)	-0,8454	-0,8454	-0,8454
(2095)	-0,8454	-0,8454	-0,8454
(2123)	-0,8454	-0,8454	-0,8454
(2125)	-0,8454	-0,8454	-0,8454
(668)	-0,69282	-0,85769	-0,69282
a-aminoacid derivative (771)	-0,8454	-0,86603	-0,8454
Endocannabinoid (2088)	-0,8454	-0,86603	-0,8454
Macrotetrolide (1062)	-0,8454	-0,86603	-0,8454
Endocannabinoid (1951)	-0,47434	-0,94868	-0,47434
Dibutoxyethoxyethyl adipate (826)	-0,47434	-0,94868	-0,47434
Dibutoxyethoxyethyl adipate (825)	-0,80408	-0,95266	-0,80408
Adipate related (807)	-0,8454	-0,9649	-0,8454
Adipate related (808)	-0,8454	-0,9649	-0,8454
Dibutoxyethoxyethyl adipate (823)	-0,8454	-0,9649	-0,8454
Glycosilated long-chain polyketide (959)	-0,8454	-0,9649	-0,8454
Polyol fatty acid (516)	-0,8454	-0,9649	-0,8454
(853)	-0,86603	-0,9649	-0,86603

EtAcFr: ethyl acetate fraction of CvExt. ButFr: butanolic fraction of CvExt. HEtFr: hydroethanolic fraction of CvExt. Full LC-MS/MS dataset can be checked at https://massive.ucsd.edu/ProteoSAFe/dataset.jsp?accession=MSV000099903.

Supplementary Table S6: Complete Spearman correlation analysis of anti-PCO activity in the most active fractions of *Cissus verticillata* leaf hydroethanolic extract (CvExt) in negative mode (ESI^-).

Compound	EtAcFr	ButFr	HEtFr
(1)	0,94868	0,94868	0,47434
(10)	0,15811	0,15811	0,15811
(101)	0,48245	0,48245	0,9649
(102)	0,9649	0,9113	0,48245
(105)	0,48245	0,48245	0,9649
(106)	0,48245	0,48245	0,9649
(108)	0,48245	0,48245	0,9649
(109)	0,48245	0,48245	0,9649
(11)	-0,57975	-0,47434	-0,47434
(110)	0,9649	0,53606	0,9649
(111)	0,9649	0,9649	0,64327
(115)	0,53606	0,48245	0,9113
(117)	0,9649	0,48245	0,64327
(118)	0,48245	0,48245	0,48245
(119)	0,9649	0,9113	0,48245
(122)	0,9649	0,53606	0,69687
(123)	0,9649	0,9649	0,9083
(124)	0,85769	0,48245	0,9649
(125)	0,53606	0,48245	0,75048
(127)	0,9649	0,9649	0,9649
(128)	0,64327	0,48245	0,48245
(129)	0,48245	0,48245	0,9649
(13)	0,95266	0,95266	0,47633
(130)	0,48245	0,48245	0,9649
(131)	0,9649	0,48245	0,48245
(132)	0,48245	0,48245	0,9649
(134)	0,48245	0,48245	0,9649
(135)	0,48245	0,48245	0,9649
(136)	0,48245	0,48245	0,9649
(14)	0,95266	0,95266	0,47633
(140)	0,53606	0,48245	0,9649
(142)	0,69687	0,69687	0,58966
(143)	0,48245	0,48245	0,9649
(144)	0,48245	0,48245	0,8454
(147)	0,48245	0,48245	0,9649
(149)	0,94868	0,47434	0,47434
(15)	0,94868	0,94868	0,47434
(150)	0,69687	0,48245	0,86603

Compound	EtAcFr	ButFr	HEtFr
(151)	0,64327	0,64327	0,9649
(153)	0,64327	0,64327	0,9649
(154)	0,9649	0,64327	0,75048
(155)	0,9649	0,48245	0,9113
(156)	0,48245	0,48245	0,9649
(157)	0,48245	0,48245	0,80408
(16)	0,94868	0,89598	0,89598
(160)	0,9649	0,58966	0,58966
(161)	0,9649	0,64327	0,48245
(165)	0,9649	0,48245	0,53606
(168)	0,48245	0,48245	0,9649
(169)	0,48245	0,48245	0,64327
(17)	0,94868	0,94868	0,94868
(170)	0,9113	0,48245	0,64327
(171)	0,48245	0,48245	0,8454
(173)	0,48245	0,48245	0,9649
(174)	0,48245	0,48245	0,75048
(175)	0,48245	0,48245	0,9649
(178)	0,9649	0,48245	0,53606
(18)	0,9649	0,9649	0,48245
(181)	0,47434	0,47434	0,68516
(183)	0,48245	0,48245	0,75056
(185)	0,48245	0,48245	0,8454
(186)	0,48245	0,48245	0,9649
(188)	0,48245	0,48245	0,8454
(19)	0,94868	0,94868	0,47434
(190)	0,48245	0,48245	0,9649
(193)	0,48245	0,48245	0,9649
(194)	0,48245	0,48245	0,9649
(195)	0,48245	0,48245	0,9649
(196)	0,48245	0,48245	0,9083
(198)	0,48245	0,48245	0,9649
(2)	0,8454	0,9649	0,48245
(20)	0,95266	0,95266	0,47633
(200)	0,9649	0,48245	0,64327
(201)	0,48245	0,48245	0,8454
(206)	0,9649	0,48245	0,9649
(207)	0,48245	0,48245	0,9649
(208)	0,48245	0,48245	0,8454
(209)	0,48245	0,48245	0,9083
(21)	0,9649	0,86603	0,48245
(210)	0,48245	0,48245	0,9083
(211)	0,53606	0,48245	0,9649
(213)	0,48245	0,48245	0,9649

Compound	EtAcFr	ButFr	HEtFr
(214)	0,48245	0,48245	0,8454
(216)	0,37048	0,37048	0,57735
(217)	0,48245	0,48245	0,9649
(22)	0,9649	0,9649	0,48245
(220)	0,48245	0,48245	0,8454
(222)	0,48245	0,48245	0,8454
(223)	0,9649	0,48245	0,9649
(224)	0,48245	0,48245	0,9649
(226)	0,48245	0,48245	0,86603
(229)	0,48245	0,48245	0,9649
(23)	-0,26352	-0,47434	-0,36893
(230)	0,48245	0,48245	0,9649
(232)	0,48245	0,48245	0,9649
(233)	0,48245	0,48245	0,8454
(234)	0,48245	0,48245	0,9649
(235)	0,47434	0,47434	0,48245
(236)	0,48245	0,48245	0,9649
(238)	0,48245	0,48245	0,9649
(239)	0,48245	0,48245	0,9649
(24)	0,9649	0,9083	0,48245
(241)	0,48245	0,48245	0,8454
(243)	0,48245	0,48245	0,8454
(244)	0,48245	0,48245	0,8454
(245)	0,48245	0,48245	0,8454
(249)	0,48245	0,48245	0,8454
(25)	0,9649	0,9649	0,48245
(250)	0,48245	0,48245	0,86603
(251)	0,48245	0,48245	0,86603
(253)	0,48245	0,48245	0,9649
(259)	0,48245	0,48245	0,9649
(26)	0,9649	0,86603	0,48245
(260)	0,48245	0,48245	0,8454
(261)	0,48245	0,48245	0,8454
(263)	0,48245	0,48245	0,8454
(265)	0,47434	0,47434	0,48245
(266)	0,48245	0,48245	0,8454
(267)	0,48245	0,48245	0,8454
(268)	0,48245	0,48245	0,8454
(27)	0,94868	0,94868	0,47434
(270)	0,48245	0,48245	0,8454
(272)	0,48245	0,48245	0,8454
(273)	0,48245	0,48245	0,8454
(274)	0,26352	0,26352	0,26803
(276)	0,48245	0,48245	0,8454

Compound	EtAcFr	ButFr	HEtFr
(279)	0,48245	0,48245	0,86603
(28)	0,9649	0,9649	0,48245
(280)	0,85769	0,48245	0,9649
(281)	0,48245	0,48245	0,8454
(282)	0,48245	0,48245	0,8454
(283)	0,48245	0,48245	0,8454
(285)	0,48245	0,48245	0,8454
(288)	0,48245	0,48245	0,8454
(289)	0,48245	0,48245	0,8454
(29)	0,9649	0,9649	0,48245
(290)	0,48245	0,48245	0,8454
(293)	0,48245	0,48245	0,8454
(294)	0,48245	0,48245	0,8454
(296)	0,48245	0,48245	0,8454
(298)	0,48245	0,48245	0,8454
(299)	0,47434	0,47434	0,48245
(3)	0,94868	0,94868	0,47434
(30)	0,48245	0,48245	0,9649
(301)	0,48245	0,48245	0,8454
(302)	0,48245	0,48245	0,8454
(303)	0,48245	0,48245	0,8454
(304)	0,48245	0,48245	0,8454
(305)	0,9113	0,48245	0,9649
(306)	0,48245	0,48245	0,8454
(307)	0,48245	0,48245	0,8454
(308)	0,48245	0,48245	0,8454
(309)	0,48245	0,48245	0,9083
(310)	0,48245	0,48245	0,8454
(312)	0,75048	0,48245	0,9649
(314)	0,48245	0,48245	0,8454
(315)	0,48245	0,48245	0,8454
(319)	0,48245	0,48245	0,8454
(320)	0,48245	0,48245	0,8454
(321)	0,58966	0,48245	0,9649
(322)	0,48245	0,48245	0,8454
(323)	0,47434	0,47434	0,48245
(325)	0,48245	0,48245	0,8454
(326)	0,48245	0,48245	0,8454
(327)	0,48245	0,48245	0,8454
(329)	0,48245	0,48245	0,8454
(33)	0,9649	0,9649	0,48245
(330)	0,48245	0,48245	0,8454
(331)	0,48245	0,48245	0,8454
(334)	0,48245	0,48245	0,8454

Compound	EtAcFr	ButFr	HEtFr
(335)	0,33029	0,38534	0,64952
(337)	0,48245	0,48245	0,8454
(339)	0,48245	0,48245	0,8454
(34)	0,9649	0,9649	0,48245
(341)	0,48245	0,48245	0,8454
(342)	0,48245	0,48245	0,86603
(344)	0,48245	0,48245	0,8454
(345)	0,33029	0,33029	0,64952
(346)	0,48245	0,48245	0,8454
(348)	-0,47434	-0,47434	-0,48245
(349)	0,48245	0,48245	0,8454
(35)	0,64327	0,48245	0,9649
(350)	0,47633	0,47633	0,69282
(353)	0,48245	0,48245	0,8454
(355)	0,47434	0,57975	0,48245
(358)	0,17321	0,17321	0,43301
(36)	0,9649	0,9649	0,48245
(362)	0,48245	0,48245	0,8454
(363)	0,48245	0,48245	0,8454
(365)	-0,94868	-0,57801	-0,57801
(373)	-0,48245	-0,48245	-0,48245
(374)	0,17321	0,2818	0,43301
(379)	0,26352	0,47434	0,26803
(38)	0,9649	0,9649	0,48245
(380)	-0,9649	-0,8454	-0,8454
(381)	0,48245	0,48245	0,8454
(383)	0,48245	0,75048	0,8454
(387)	-0,47434	-0,57975	-0,48245
(392)	0,26803	0,37524	0,40705
(394)	0,80829	0,48245	0,8454
(395)	-0,95266	-0,69282	-0,69282
(399)	0,9649	0,9649	0,8454
(4)	0,95266	0,95266	0,47633
(400)	-0,94868	-0,47434	-0,48245
(402)	-0,8454	-0,9083	-0,8454
(404)	-0,95266	-0,69282	-0,69282
(405)	-0,94868	-0,47633	-0,48245
(408)	-0,9649	-0,8454	-0,8454
(41)	0,9649	0,58966	0,9649
(413)	0,85769	0,48245	0,8454
(414)	-0,94868	-0,47434	-0,48245
(417)	-0,89598	-0,63246	-0,48245
(420)	0,53606	0,48245	0,8454
(423)	-0,79057	-0,47434	-0,48245

**Compound**	**EtAcFr**	**ButFr**	**HEtFr**
(424)	0,9083	0,48245	0,8454
(425)	-0,47434	-0,94868	-0,48245
(428)	-0,94868	-0,57801	-0,57801
(429)	-0,94868	-0,47434	-0,48245
(43)	0,48245	0,48245	0,9649
(430)	-0,9649	-0,8454	-0,8454
(433)	0,8454	0,48245	0,8454
(434)	-0,79057	-0,48245	-0,48245
(435)	-0,63246	-0,68516	-0,48245
(437)	-0,47434	-0,47434	-0,48245
(439)	-0,94868	-0,47434	-0,48245
(440)	0,94868	0,57975	0,68516
(443)	-0,94868	-0,58218	-0,48245
(445)	-0,94868	-0,48245	-0,48245
(446)	0,85769	0,58966	0,8454
(448)	-0,48245	-0,48245	-0,47434
(449)	0,62622	0,44039	0,64952
(451)	0,47434	0,47434	0,48245
(452)	-0,9649	-0,8454	-0,8454
(454)	-0,94868	-0,48245	-0,48245
(455)	0,94868	0,47434	0,82035
(457)	-0,9649	-0,8454	-0,8454
(458)	-0,86603	-0,8454	-0,8454
(46)	0,94868	0,68516	0,47434
(461)	0,80829	0,48245	0,8454
(462)	0,94868	0,47434	0,47633
(463)	-0,9649	-0,8454	-0,8454
(465)	-0,63246	-0,94868	-0,47434
(467)	0,94868	0,47434	0,47434
(470)	0,64952	0,33029	0,64952
(471)	-0,94868	-0,48245	-0,48245
(472)	-0,94868	-0,47434	-0,48245
(473)	-0,94868	-0,48245	-0,48245
(474)	-0,94868	-0,47434	-0,48245
(478)	0,94868	0,47434	0,73786
(479)	-0,94868	-0,47633	-0,48245
(48)	0,48245	0,48245	0,48245
(480)	-0,94868	-0,47434	-0,48245
(485)	-0,94868	-0,48245	-0,48245
(488)	0,47434	0,47434	0,48245
(49)	0,9649	0,48245	0,48245
(490)	-0,68516	-0,52705	-0,48245
(491)	-0,84327	-0,57801	-0,57801
(492)	-0,94868	-0,47434	-0,48245

Compound	EtAcFr	ButFr	HEtFr
(493)	0,47434	0,47434	0,48245
(494)	0,94868	0,47434	0,94868
(495)	-0,94868	-0,48245	-0,48245
(496)	-0,94868	-0,48245	-0,48245
(5)	0,94868	0,94868	0,47434
(500)	-0,9649	-0,8454	-0,8454
(502)	0,94868	0,47434	0,94868
(504)	0,47434	0,47434	0,48245
(505)	-0,94868	-0,57801	-0,57801
(508)	-0,57801	-0,66157	-0,57801
(509)	-0,94868	-0,47434	-0,48245
(51)	0,48245	0,48245	0,85769
(510)	0,47434	0,47434	0,47434
(511)	-0,8454	-0,8454	-0,8454
(513)	0,47434	0,47434	0,48245
(514)	0,94868	0,52705	0,47434
(516)	0,062622	0,16514	0,072169
(517)	-0,94868	-0,47633	-0,48245
(518)	-0,94868	-0,48245	-0,48245
(52)	0,9649	0,48245	0,48245
(520)	-0,94868	-0,48245	-0,48245
(521)	0,94868	0,47434	0,48245
(523)	0,94868	0,68516	0,47434
(526)	-0,47434	-0,48245	-0,48245
(527)	-0,47434	-0,48245	-0,48245
(530)	0,94868	0,79057	0,47434
(533)	-0,8454	-0,86603	-0,86603
(534)	0,94868	0,73786	0,57975
(535)	-0,63246	-0,94868	-0,94868
(536)	-0,48245	-0,48245	-0,48245
(537)	-0,48245	-0,48245	-0,48245
(538)	-0,8454	-0,8454	-0,8454
(539)	-0,48245	-0,48245	-0,48245
(540)	0,94868	0,84327	0,52705
(541)	-0,47434	-0,89598	-0,79057
(542)	-0,94868	-0,48245	-0,48245
(543)	-0,94868	-0,48245	-0,48245
(544)	-0,94868	-0,57801	-0,57801
(545)	0,43301	0,34641	0,43301
(546)	-0,9649	-0,8454	-0,8454
(547)	-0,8454	-0,8454	-0,8454
(549)	-0,47434	-0,48245	-0,48245
(550)	0,94868	0,47434	0,89598
(551)	-0,85769	-0,69282	-0,69282

Compound	EtAcFr	ButFr	HEtFr
(552)	-0,89598	-0,52926	-0,48245
(553)	-0,8454	-0,8454	-0,8454
(554)	-0,8454	-0,8454	-0,8454
(555)	0,8454	0,48245	0,8454
(559)	0,94868	0,52705	0,68516
(560)	0,94868	0,47434	0,89598
(563)	0,9649	0,48245	0,8454
(564)	-0,94868	-0,47633	-0,48245
(566)	-0,47434	-0,89598	-0,47434
(567)	0,95266	0,63511	0,69282
(568)	0,47434	0,84327	0,48245
(569)	-0,8454	-0,8454	-0,8454
(57)	0,86603	0,9649	0,48245
(570)	0,94868	0,47434	0,79057
(571)	0,94868	0,47434	0,73786
(572)	0,8454	0,48245	0,8454
(573)	-0,48245	-0,85769	-0,80408
(574)	-0,8454	-0,8454	-0,8454
(575)	-0,8454	-0,8454	-0,8454
(576)	-0,8454	-0,8454	-0,8454
(579)	-0,94868	-0,47434	-0,48245
(580)	-0,94868	-0,48245	-0,48245
(581)	-0,63246	-0,94868	-0,48245
(582)	-0,79057	-0,47434	-0,48245
(583)	0,55048	0,33029	0,66058
(6)	0,9649	0,9649	0,48245
(60)	0,9649	0,9649	0,48245
(62)	0,85769	0,48245	0,85769
(65)	0,9649	0,9649	0,53606
(67)	0,9649	0,9649	0,58966
(68)	0,48245	0,48245	0,9649
(7)	0,9649	0,9649	0,48245
(73)	0,9649	0,9083	0,48245
(77)	0,9649	0,58966	0,48245
(8)	0,9649	0,9649	0,48245
(80)	0,48245	0,48245	0,9083
(81)	0,48245	0,48245	0,9649
(82)	0,48245	0,48245	0,9649
(84)	0,48245	0,48245	0,80408
(85)	0,48245	0,48245	0,9649
(86)	0,94868	0,47434	0,47434
(88)	0,48245	0,48245	0,9649
(89)	0,9649	0,9649	0,53606
(91)	0,9649	0,9649	0,48245

Compound	EtAcFr	ButFr	HEtFr
(92)	0,48245	0,48245	0,9649
(95)	0,53606	0,48245	0,85769
(99)	0,48245	0,48245	0,9649
16-Hydroxyhexadecanoic acid (524)	-0,9649	-0,8454	-0,8454
1-O-(4-Coumaroyl)-beta-D-glucose (104)	0,48245	0,48245	0,9649
2-({6-O-[(2E)-3-(4-Hydroxyphenyl)-2-propenoyl]-beta-D-glucopyranosyl}oxy)-3-phenylacrylic acid (277)	0,48245	0,48245	0,8454
2-(3,4-dihydroxyphenyl)-5,8-dihydroxy-7-methoxy-3-[(2S,3R,4R,5R,6S)-3,4,5-trihydroxy-6-methyloxan-2-yl]oxychromen-4-one (231)	0,48245	0,48245	0,8454
3-Hydroxycinnamic acid (197)	0,48245	0,48245	0,86603
3-O-Feruloylquinic acid (163)	0,48245	0,48245	0,75048
4-oxododecanedioic acid (252)	0,26352	0,26352	0,35781
5-trans-Prostaglandin D2 related (406)	-0,94868	-0,58218	-0,48245
6,7-Dihydroxycoumarin (139)	0,48245	0,48245	0,9649
7-(geranyloxy)coumarin (450)	0,86603	0,48245	0,8454
9,12,13,TriHODE related (376)	0,48245	0,48245	0,8454
9S-HpOTrE related (397)	-0,9649	-0,8454	-0,8454
Afzelin (255)	0,48245	0,48245	0,8454
Afzelin (256)	0,48245	0,48245	0,8454
Afzelin (257)	0,48245	0,48245	0,9649
Afzelin (258)	0,47434	0,47434	0,94868
Afzelin related (262)	0,48245	0,48245	0,8454
Apigenin_Hex_Hex (121)	0,53606	0,48245	0,9649
Apigenin_Pen_Hex (148)	0,48245	0,48245	0,9649
Apigenin_Pen_Hex (158)	0,53606	0,48245	0,9649
Benzoic acid + 2O_ O-Hex (63)	0,9649	0,9649	0,48245
Chlorogenic acid (133)	0,48245	0,48245	0,9649
Chlorogenic acid (159)	0,48245	0,48245	0,86603
Chlorogenic acid (98)	0,48245	0,48245	0,9649
Chlorogenic acid related (145)	0,47434	0,47434	0,94868
Chlorogenic acid related (177)	0,47633	0,47633	0,95266
Citric acid (39)	0,86603	0,8454	0,48245
Citric acid (45)	0,9649	0,9649	0,48245
Corchoionoside C (179)	0,47434	0,47434	0,94868
Coumaric acid (103)	0,48245	0,48245	0,9649
Coumaric acid (184)	0,48245	0,64327	0,8454
Coumaric acid (212)	0,48245	0,48245	0,8454
Coumaroyl + C6H9O8 (116)	0,9649	0,9649	0,58966
Coumaroyl + C6H9O8 (78)	0,9649	0,9649	0,48245
Coumaroyl + C6H9O8 (90)	0,9649	0,9649	0,48245
Cyanidin-3-O-rutinoside (205)	0,48245	0,48245	0,9649
Dihydrokaempferol (271)	0,48245	0,9649	0,8454

Compound	EtAcFr	ButFr	HEtFr
Echinocystic acid (561)	-0,94868	-0,48245	-0,48245
Ethiprole related (482)	-0,84327	-0,63246	-0,47434
FA 18:2+3O (360)	0,47434	0,47434	0,47434
Fipronil sulfone (499)	-0,84327	-0,63246	-0,47434
Gentisate (114)	0,48245	0,48245	0,9649
Gentisate (72)	0,48245	0,48245	0,63305
Glc-Glc-octadecatrienoyl-sn-glycerol (431)	0,47434	0,47434	0,48245
Glc-Glc-octadecatrienoyl-sn-glycerol (441)	0,47434	0,47434	0,48245
Homoarginin (9)	0,9649	0,9649	0,48245
Homoaspartic acid (12)	0,79057	0,79057	0,47434
Isochlorogenic acid B related (94)	0,48245	0,48245	0,9649
Kaji-ichigoside F1 (352)	0,48245	0,48245	0,8454
Kinic acid (146)	0,48245	0,48245	0,9649
Kinic acid (176)	0,48245	0,48245	0,9649
Kinic acid (97)	0,48245	0,48245	0,9649
methyl (2E,4E,8E)-7,13-dihydroxy-4,8,12-trimethyltetradeca-2,4,8-trienoate (475)	-0,94868	-0,57801	-0,57801
methyl (2E,4E,8E)-7,13-dihydroxy-4,8,12-trimethyltetradeca-2,4,8-trienoate (483)	-0,89598	-0,48245	-0,48245
Myricetin 3-O-rutinoside (152)	0,48245	0,48245	0,8454
Myricetin-3-O-galactoside (164)	0,48245	0,48245	0,8454
Myricitrin (189)	0,48245	0,48245	0,8454
Myricitrin (191)	0,47434	0,47434	0,94868
Myricitrin (192)	0,48245	0,48245	0,9649
Myricitrin (278)	0,48245	0,48245	0,8454
Myricitrin (284)	0,48245	0,48245	0,8454
Neochlorogenic acid (74)	0,48245	0,48245	0,80408
Pantothenic acid-B5 (64)	0,48245	0,48245	0,9649
PI(18:1/0:0) (444)	0,9649	0,48245	0,8454
Quercitrin (225)	0,48245	0,48245	0,9083
Quercitrin (227)	0,47434	0,47434	0,94868
Quercitrin (228)	0,48245	0,48245	0,8454
Rutin (182)	0,48245	0,48245	0,9649
Taxifolin (219)	0,33029	0,38534	0,64952
Tryptophan (79)	0,48245	0,48245	0,9649
Uracil arabinoside (47)	0,48245	0,48245	0,64327
Usolic/oleanolic acid (529)	-0,8454	-0,8454	-0,8454

EtAcFr: ethyl acetate fraction of CvExt. ButFr: butanolic fraction of CvExt. HEtFr: hydroethanolic fraction of CvExt. Full LC-MS/MS dataset can be checked at https://massive.ucsd.edu/ProteoSAFe/dataset.jsp?accession=MSV000099903.

Supplementary Table S7: LC-MS/MS processing parameters utilized on the MZMine software.

**Mass detection**	
MS 1	1E3
MS 2	5E1
**Chromatogram builder**	
Minimum consecutive scans	4
Minimum intensity for consecutive scans	1E3
Minimum absolute height	3E3
m/z tolerance	20 ppm
**Smoothing**	
Smoothing algorithm	Savitzky Golay
Retention time smoothing	5
**Local minimum resolver**	
Dimension	Retention time
Chromatographic threshold	89.3%
Minimum search range RT/mobility (absolute)	0.05
Minimum absolute height	3E3
Min ratio of peak top/edge	1.8
Peak duration range	0 – 1.51
Minimum scans	4
**13C isotope filter**	
m/z tolerance (intra-sample)	3 ppm
Retention time tolerance	0.04 minutes
Monotonic shape	Yes
Maximum charge	2
Representative isotope	Most intense
Never remove feature with MS2	Yes
**Join aligner**	
m/z tolerance	8 ppm
Weight for m/z / RT	3 / 1
Retention time tolerance	0.1 minutes
**Peak finder**	
Intensity tolerance	30%
m/z tolerance	20 ppm
Retention time tolerance	0.1 minutes
Minimum scans (data points)	2
**Duplicate peak filter**	
Filter mode	New average
m/z tolerance	1.5 ppm
RT tolerance	0.04 minutes
**Correlation grouping (metaCorrelate)**	
RT tolerance	0.06 minutes
Intensity threshold for correlation	1E4
Feature shape correlation	
Min data points	5
Min data points on edge	2
**Correlation grouping (metaCorrelate)**	
Measure	Pearson
Min shape correlation	85%
Feature height correlation	
Minimum samples	2
Measure	Pearson
Min correlation	70%

**Ion identity networking**	
m/z tolerance	3 ppm
Check	All features
Ion identity library	Adducts: -H_2O; NH4; Na; K; Cl; FA.

Supplementary Table S8: Calculation parameters of SIRIUS for *in silico* annotation. Computations were made in positive and negative mode separately.

**SIRIUS**	Enabled
**Instrument**	Q-TOF
**MS2 mass accuracy (ppm)**	10
**Fix formula for detected lipid**	Yes
**Fallback adducts**	[M+H]+ / [M-H]-
**Molecular formula generation**	De novo + bottom up
**Perform de novo below m/z**	400
**Apply element filter to**	De novo
**Allowed elements**	H, C, N, O
**ZODIAC**	Enabled
**CSI:FingerID - CANOPUS**	Enabled (with score threshold enabled)
**CSI:FingerID Structure database search**	Enabled
**PubChem as fallback**	Enabled
**Confidence mode**	Approximate
**Search DB**	Bio only
**MSNovelist**	Disabled
**Advanced settings**	Default

Figure S1: Reaction velocity ratios plotted against the concentrations of the standard antioxidants or CvExt (sample) in the crocin bleaching assay ($ROO^{\bullet}$ scavenging assay). **(A)** Quercetin; **(B)** Trolox; **(C)** CvExt.

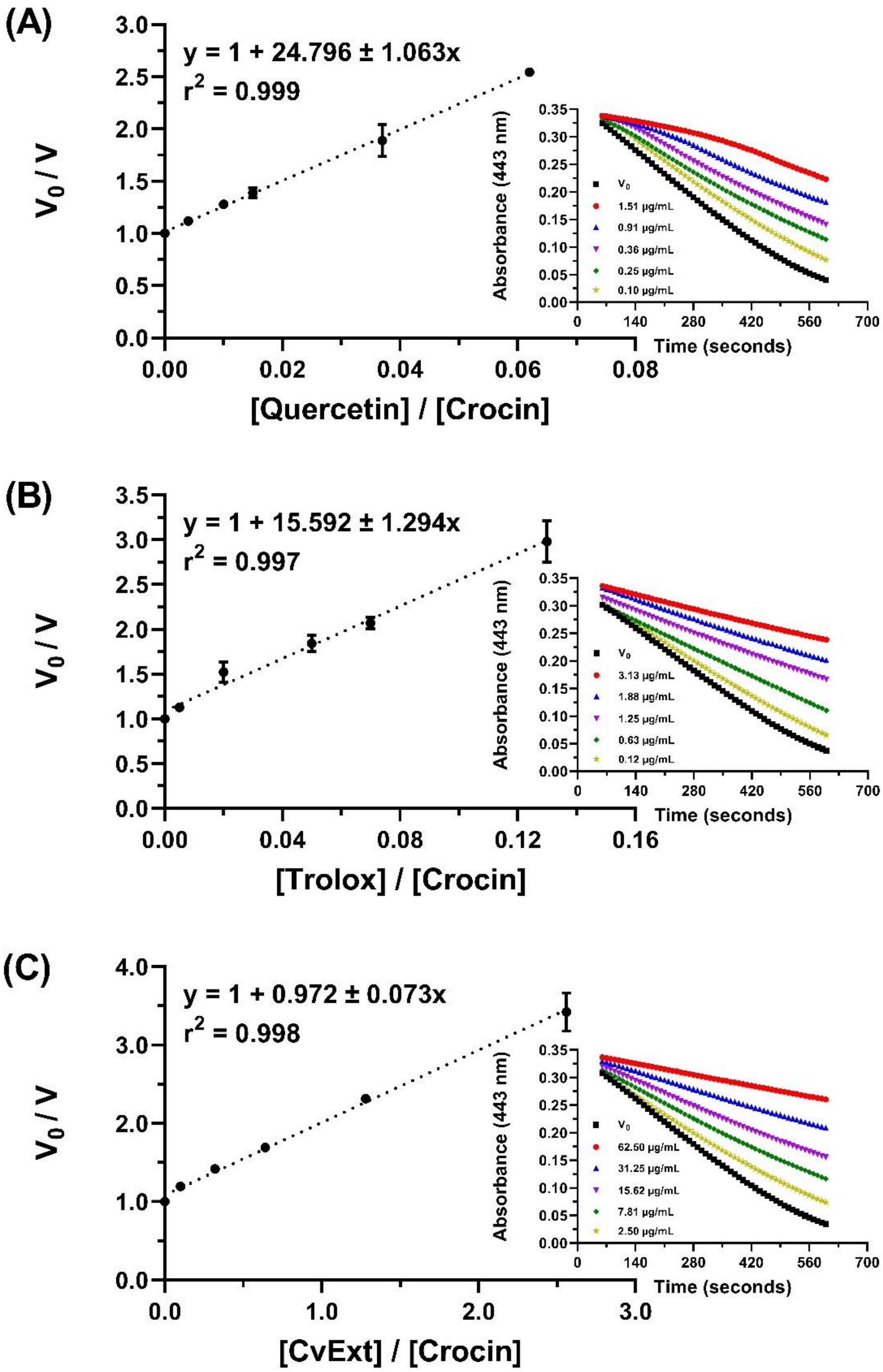

[C], crocin concentration; [S], sample concentration; V_0, velocity in the absence, and V, velocity in the presence of various concentrations of samples. The inset graphs show the decrease in the absorbance of crocin, at 443 nm. CvExt: Cissus verticillata leaf hydroethanolic extract.

Figure S2: Capacity of the standard antioxidants or CvExt (sample) to scavenge $O_2^{\bullet-}$. **(A)** Quercetin; **(B)** Trolox; **(C)** CvExt.

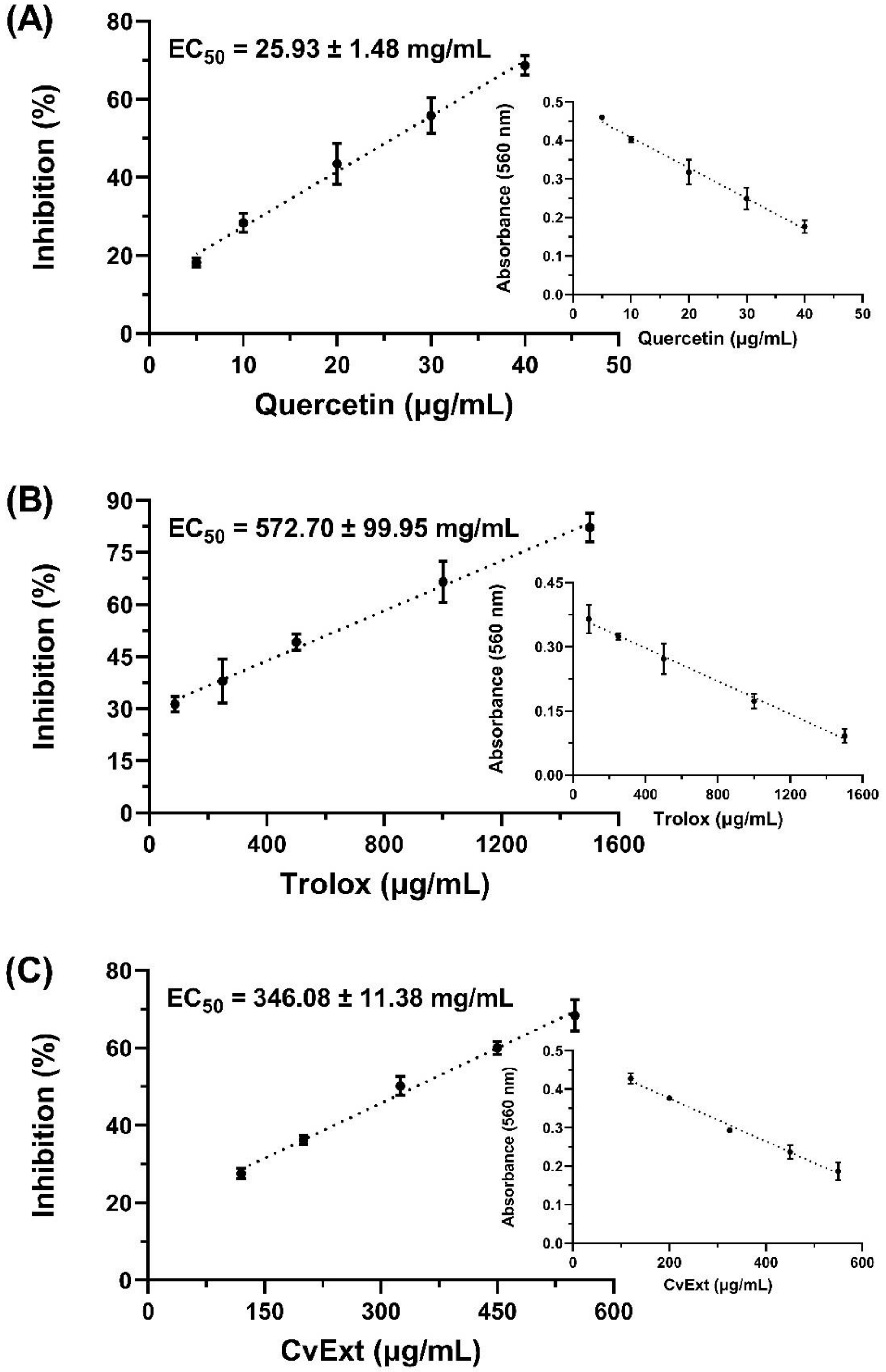

The inset graphs show the mean absorbance of the formazan (generated from nitrotetrazolium blue reduction) at 560 nm. CvExt: *Cissus verticillata* leaf hydroethanolic extract.

Figure S3: Absorption spectra of incubations containing BSA + CvExt after 30 days **(A)** or BSA + CvExt after 4 days **(B)**.

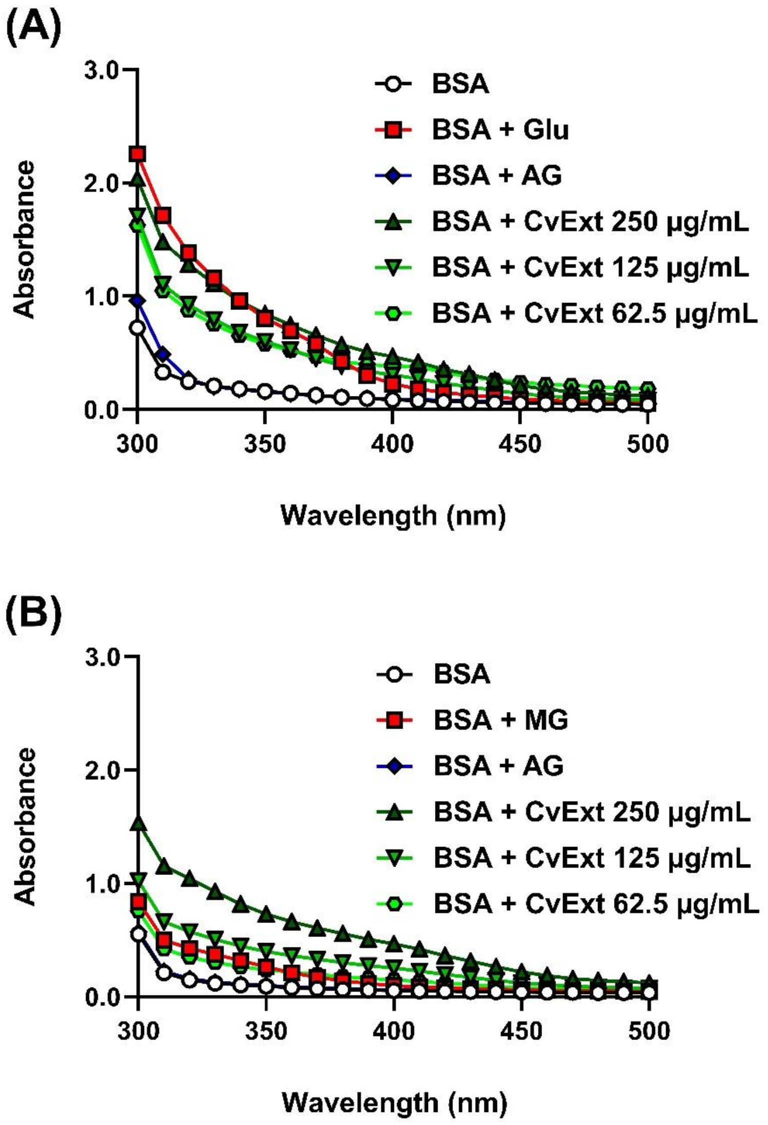

BSA: bovine serum albumin. Glu: glucose. MG: methylglyoxal. AG: aminoguanidine. CvExt: *Cissus verticillata* leaf hydroethanolic extract.

Figure S4: Absorption spectra of incubations containing BSA + glucose + CvExt after 30 days **(A)** or BSA + MG + CvExt after 4 days **(B)**.

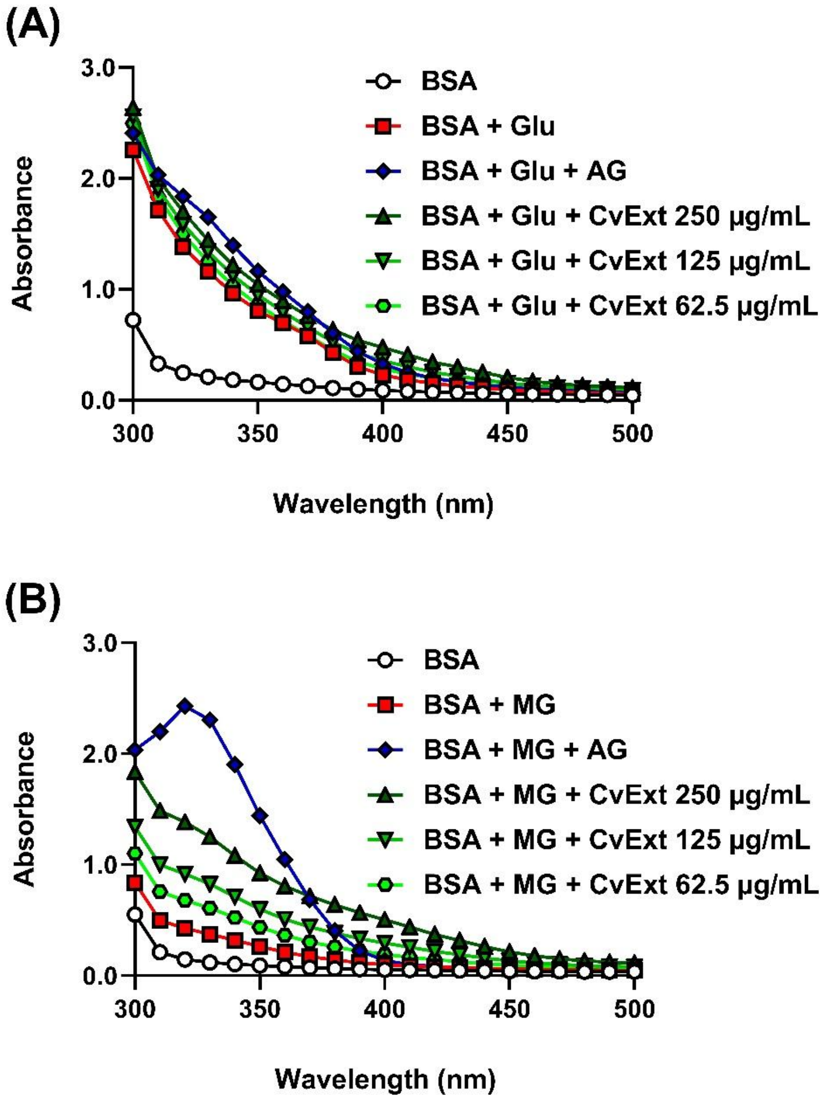

BSA: bovine serum albumin. Glu: glucose. MG: methylglyoxal. AG: aminoguanidine. CvExt: *Cissus verticillata* leaf hydroethanolic extract.
